# Supplementary material for: Multicountry research on comprehensive abortion policy implementation in Latin America: a mixed-methods study protocol
Source: BMJ Open. 2024 Jan 20;14(1):e073617. doi: 10.1136/bmjopen-2023-073617 (PMC10806677; doi:10.1136/bmjopen-2023-073617)
Supplement: Author's manuscript [file bmjopen-2023-073617.draft_revisions.pdf]

BMJ Open is committed to open peer review. As part of this commitment we make the peer review history of every article we publish publicly available.

When an article is published we post the peer reviewers' comments and the authors' responses online. We also post the versions of the paper that were used during peer review. These are the versions that the peer review comments apply to.

The versions of the paper that follow are the versions that were submitted during the peer review process. They are not the versions of record or the final published versions. They should not be cited or distributed as the published version of this manuscript.

BMJ Open is an open access journal and the full, final, typeset and author-corrected version of record of the manuscript is available on our site with no access controls, subscription charges or pay-per-view fees (http://bmjopen.bmj.com).

If you have any questions on BMJ Open's open peer review process please email info.bmjopen@bmj.com

BMJ Open

Multi-country research on comprehensive abortion policy implementation in Latin America: a mixed-methods study protocol

Journal:	*BMJ Open*
Manuscript ID	bmjopen-2023-073617
Article Type:	Protocol
Date Submitted by the Author:	21-Mar-2023
Complete List of Authors:	Gialdini, Celina; Centro Rosarino de Estudios Perinatales,<br>Ramón Michel, Agustina; CEDES<br>Romero, Mariana; CEDES; CONICET<br>Ramos, Silvina; CEDES<br>Carroli, Guillermo; CREP<br>Carroli, Berenise; CREP<br>Gomez Ponce de León, Rodolfo; Pan American Health Organization, CLAP WRH<br>Vila Ortiz, Mercedes; CREP<br>Lavelanet, Antonella ; WHO, ; World Health Organization
Keywords:	PUBLIC HEALTH, Health policy < HEALTH SERVICES ADMINISTRATION & MANAGEMENT, SEXUAL MEDICINE

SCHOLARONE™
Manuscripts

Multi-country research on comprehensive abortion policy implementation in Latin America: a mixed-methods study protocol

Authors: Gialdini, C.*[1], Ramón Michel, A.[2], Romero, M.[2,3], Ramos, S.[2], Carroli, G.[1], Carroli, B. [1], Gomez Ponce de León, R.[4], Vila Ortiz, M. [1], Lavelanet, A[5].

*Corresponding author, cgialdini@crep.org.ar, Centro Rosarino de Estudios Perinatales

Abstract

Introduction

Access to comprehensive abortion care could prevent the death of between 13,865 and 38,940 women and the associated morbidity of five million women worldwide. There have been some important improvements in Latin America in terms of laws and policies on abortion. However, the predominant environment is still restricted and many women, adolescents, and girls still face multiple barriers to exercise their reproductive rights. This research will systematically assess comprehensive abortion policies in five Latin American countries (Argentina, Colombia, Honduras, Mexico, and Uruguay). The aim is to identify barriers, facilitators, and strategies to the implementation of abortion policies, looking at four key dimensions −regulatory framework, abortion policy dynamics, abortion service delivery, and health system and health outcomes indicators− to draw cross-cutting lessons learned to improve current implementation and inform future safe abortion policy development.

Methods and analysis

A mixed-method design will be used in the five countries to address the four dimensions through the availability, accessibility, acceptability, and quality of care model. The data collection tools include desk reviews and semi-structured interviews with key actors. Analysis will be performed using thematic analysis and stakeholder analysis. A regional synthesis exercise will be conducted to draw lessons on barriers, facilitators, and the strategies to deal with them.

Ethical considerations

The project has been approved by the World Health Organisation Research Ethics Review Committee and by the local Research Ethics Committees. Informed consent will be obtained. Data will be treated with careful attention to protecting privacy and confidentiality.

Dissemination

Findings from the study will be disseminated through a multipurpose strategy to target diverse audiences to foster the use of the study findings to inform the public debate agenda and policy implementation at national level. The strategy will include academic, advocacy and policy arenas and actors: peer-reviewed publication, national and regional dissemination workshops.

Strengths and limitations

- The study encompasses five countries with a variety of legal environments regarding abortion. This will show different contexts while looking at common patterns regarding barriers, facilitators, and strategies.
- From all complex social, legal, cultural, and ethical factors shaping abortion care and policies, this research will focus on four, using the AAAQ framework. In this process, women's voices are not included directly. However, women's organizations, which usually have relevant and updated information about women's experiences with abortion, are participants in this study as stakeholders.
- This study will focus on factors influencing implementation and will not analyse how these factors might correspond to other aspects, such as the evaluation of the policies, the estimation of satisfaction with the care provided or the magnitude and severity of abortion-related complications in relation to policy implementation.
- Data will be collected through standardized instruments. However, there are challenges in providing a systematic comparison of heterogeneous countries. Moreover, the samples in this study may not reflect the heterogeneity of institutions and the proportionality of the population.
- Principal and country investigators will commit to promote capacity building activities and include junior researchers in the development of the study.

Introduction

Access to comprehensive abortion care could prevent the death of between 13,865 and 38,940 women (1) and the associated morbidity of five million women

For peer review only

worldwide. (2) Improving access to quality abortion care is an essential strategy for the provision of universal access to reproductive health and for the achievement of the United Nations Sustainable Development Goals (SDG): good health and well-being and gender inequality (SDGs 3 and 5). Women's access to sexual and reproductive rights can also contribute to reproductive empowerment, and therefore address additional SDGs such as quality education (SDG 4), sanitation and hygiene (SDG 6), decent work and economic growth (SDG 8), reduce inequalities (SDG 10) and develop peace, justice, and strong institutions (SDG 16). (3)

Since the adoption of the Programme of Action (PoA) of the International Conference on Population and Development (ICPD) 28 years ago, countries have been called to strengthen their commitment to women's health by addressing unsafe abortion and supporting a woman's right to decide. (4) Although some improvements in terms of policies on sexual and reproductive health −including law reforms− have been achieved, many women in Latin America face multiple barriers to exercise their reproductive rights. (5)

Obstacles to quality abortion access are a matter of human rights and of public health: in this region, at least 10% of all maternal deaths are a consequence of unsafe abortions and near 760,000 women are hospitalized per year due to unsafe abortion related complications, which violates their rights to life and health. (6) The obstacles faced by women, adolescents and girls when trying to access quality abortion include the interpretation and the implementation of the grounds (i.e., legal indications under which is legal to have an abortion) for having a legal abortion, the lack of access to information, stigma, restricted type of health-care providers and scarcity of facilities that can lawfully provide services, among others. (7) These barriers can disproportionately affect specific groups, such as adolescents, women that are poor, indigenous women, women living in small towns, and other women in vulnerable social conditions.

Despite the efforts and claims, abortion laws have been difficult to reform. Most Latin American countries have restrictive abortion laws, allowing legal termination of pregnancy only under certain grounds such as rape, severe fetal impairment or when the woman's life or health is at risk. (8) Currently abortion is legal without restriction

For peer review only

as to reason in the first twelve-fourteen weeks of pregnancy in Argentina, Cuba, Uruguay, and nine states of Mexico (Mexico City, Oaxaca, Hidalgo, Veracruz, Baja California, Baja California Sur, Sinaola, Guerrero and Colima). More recently the Colombian Constitutional Court has stated that abortion should be decriminalized up to 24 weeks of pregnancy.

Nevertheless, some countries have been able to improve access to quality abortion services resorting to other instruments. Court judgments and public health guidelines have been used to expand the interpretation of the circumstances under which abortion is allowed. In settings with very restrictive legal environments, like Honduras, guidelines to manage post-abortion care (PAC) have also been issued. (9)

Taking this regional context into consideration, this research will systematically look at comprehensive abortion policies in five Latin American countries: Argentina, Colombia, Honduras, Mexico, and Uruguay. The aim is to assess abortion regulations and their use in practice, the policy dynamics, the service delivery arrangements, and the abortion-related health indicators and how they are monitored and evaluated, to identify barriers and facilitators to the implementation of comprehensive abortion policy and the strategies used to address them in each country. Finally, this study will identify similarities and differences among barriers, facilitators, and strategies of policy implementation across countries, and draw lessons learned from these implementation processes.

Methods and analysis

Design and framework

This research study will look at the context, content, actors, and processes affecting the implementation and monitoring of comprehensive abortion policies and comprises five country-case studies and a regional synthesis of findings. A mixed-method design using diverse data gathering techniques and analysis strategies will assess four key dimensions of abortion policy:

- The **regulatory framework** (dimension 1)
- The **policy dynamics** (dimension 2)

For peer review only

- The **service delivery arrangements** (dimension 3)
- The **health system and health outcomes indicators** (dimension 4)

Dimension 1 (regulatory framework) will assess how and to what extent regulations −both written and in practice− pose as barriers and facilitators to quality abortion access, and the strategies to deal with them. It will address the traits and use of existing abortion related regulations. This dimension encompasses laws, resolutions, decrees, health standards and health guidelines, technical guidelines, and other official documents with or without legal status. First, a descriptive analysis of the regulatory framework will be performed, and then an examination of the practice.

Dimension 2 (policy dynamics) will assess how aspects such as agenda setting, design and implementation processes, and governance and actors' involvement, pose as barriers and facilitators of abortion policy, and how they are addressed through different strategies. Agenda setting will be assessed looking at the political and social conditions that place abortion policy on the public agenda. Design and implementation aspects will focus on the shaping of content in policy formulation and on the main challenges of implementation. Governance issues such as the capacity of health authorities to rule, reinforce and have control of the implementation of abortion policy for the provision and access to comprehensive abortion care will be assessed. The policy dynamics will be addressed from the perspective of policy makers, health services managers and women's organizations.

Dimension 3 (service delivery arrangements) will assess how health services are organised to ensure access to quality abortion care, and how barriers and facilitators are addressed by different strategies. (10-14)

Dimension 4 (health system and health outcomes indicators) will assess what information on health system and health outcomes indicators related to abortion care are monitored and evaluated. It will address the process of generating, collecting, reporting, monitoring, and evaluating health system and health outcomes indicators, and how this evaluation accounts for policy implementation progress and quality of care oversight.

These dimensions will be assessed through availability, accessibility, acceptability, and quality of care as framed in the **AAAQ model**. This framework will be used to gather and systematize the data coming from the two data collection instruments: semi-

For peer review only

structured interviews and document review. *Availability* refers to the need of having enough functioning public health and health care facilities, goods, and services. *Accessibility* refers to the need of making all those facilities, goods, and services physically accessible and affordable to everyone within the jurisdiction of the State party without discrimination. *Acceptability* refers to the need of the health system responses to be respectful of the law, medical ethics, and to be culturally appropriate and sensitive to gender and age. *Quality of care* also refers to the need for health goods and services to be scientifically and medically approved and of good quality following WHO standards. (15) Specifically, *quality of abortion care* refers to the fact that the provision of abortion services should be effective, efficient, accessible, acceptable (patient centred), equitable and safe. (1)

As a result of the appraisal of the four dimensions, **barriers and facilitators** to availability, accessibility, acceptability, and quality of care regarding abortion from abortion policy implementation will be identified.

Finally, findings from the case studies will be synthetized to draw lessons learned on cross-cutting barriers and facilitators, as well as the similarities and differences on how comprehensive abortion policies are implemented in the participant countries. These lessons learned can be used to inform future decision-making across the region and other sociocultural and geopolitical contexts. (See Fig. 1).

For this study, comprehensive abortion care includes the elements of induced abortion under legal indications as well as all the elements of post abortion care (PAC): information, counselling, treatment, contraceptive services, reproductive health services, community, and service providers' partnerships.

Fig.1. Research framework: dimensions & methods

Settings

Case-studies will be conducted in five selected countries: Argentina, Colombia, Honduras, Mexico, and Uruguay. The selection of settings addresses diversity in abortion regulations as well as diversity in countries' institutional political organization across a continuum of abortion related legal access. This aims to include countries going

For peer review only

from an absolute legal restrictive environment with only post-abortion care provision to countries with less restrictive legal status of abortion, availability of abortion guidelines, or registration and mifepristone and misoprostol availability. The selection also considers common issues on abortion policy implementation and indicators among countries.

Data collection and tools

Two data collection methods will be undertaken: desk review (for dimensions 1 and 4) and semi-structured interviews (for dimensions 1, 2, 3, and 4). The data collection process is summarized in Table 1.

Desk review

For dimension 1, a review of the texts of regulations will be undertaken. To identify and categorize abortion related regulations, a data extraction matrix will be structured in two domains:

− the abortion regulatory framework, which refers to the specific regulations and practices related to abortion.

− the legal and policy environment, which includes regulations that are not specifically about abortion but have a direct impact on access to abortion health services (e.g., laws regarding patient confidentiality, norms regarding minors' consent, norms on mental or intellectual disabilities, laws related to sexual violence).

For dimension 4, a systematic search for country-specific statistics, reports and documents will be carried out. National official reports and databases containing processes, outputs, or outcome indicators relevant to abortion care at national level will be reviewed and gaps in indicator reporting, monitoring and evaluation will be identified.

The data extraction matrix will be based, for dimension 1, on the topics included in the WHO Global Abortion Policies Database (9, 16) and in the report "*Barreras en el acceso a los abortos legales: una mirada a las regulaciones sanitarias que incluyen el uso del misoprostol*". (17) For dimension 4, a data extraction matrix structured with the AAAQ domains will serve to identify and categorize abortion related indicators. From each

For peer review only

reported indicator, data on indicator definition, method of measurement, data collection methodology and frequency, date of publication, source of information and indicator value will be collected. If data are stratified (e.g., race, gender, age, urban/rural), this information will also be collected.

Semi-structured interviews

Semi-structured interviews (18) will explore:

- how legal and regulatory instruments are used, how they pose as barriers and facilitators to abortion service provision and access and how regulations are translated into practice (dimension 1).
- the policy dynamics related to agenda setting, governance issues, financing schemes, and actors' involvement (dimension 2).
- which are the service arrangements adopted according to the different settings, human resources, and the technologies available, and which were the triggers for the model/different models currently used (dimension 3); and
- which approaches each country uses for monitoring abortion-related care and for the oversight of maternal mortality, near miss and quality of care and how monitoring and evaluation of process −and outcomes− indicators related to abortion are operationalized (dimension 4).

Interviews will be conducted by an experienced interviewer with social science or public health background with legal or programmatic knowledge. The interviews will be recorded after informed consent has been obtained and then transcribed. The interviews may be conducted in person or through digital technology.

<u>Participants</u>

For the interviews, we will use a purposive sample of twenty key informants per dimension in each country. This sample seeks to obtain a broad range of perspectives on the subject (19). The selected subjects will be key informants in the abortion policy domain, encompassing different categories: health providers, sexual and reproductive health policy decision makers, researchers, leaders of women's organizations, judicial agents, politicians, opinion leaders, etc.

For peer review only

The sample size is a target overall number, which will be adjusted during the data collection process. Twenty key informants per dimension in each country is considered a feasible sample size that should allow for variability along the different categories (see Table 1).

The identification of participants is expected to be different depending on how broad or narrow the categories or levels from where they are identified are. The criteria of variability of forums or levels aims at obtaining diversity and having those that yield the most plentiful data in the matter.

The key informants will be identified through a mapping of actors. A first round of mapping will be based on teams' expertise and networks. Narrow-level participants such as decision makers (who are few in each national abortion domain) are expected to be easily identified. For broad categories, such as health providers and activists (who are many and disperse in different settings), the selection strategy will be ultimately defined with each country's principal investigator to achieve a convenience sample that accounts for variability. There may be a second round of mapping to select knowledgeable stakeholders identified by the interviewees from the first round.

Table 1 Data collection

**ARGENTINA**	**Dimension 1**	**Dimension 2**	**Dimension 3**	**Dimension 4**	**Total**
	Desk review<br>20 interviews	20 interviews	20 interviews	Desk review<br>10 interviews	2 desk reviews<br>70 interviews
**COLOMBIA**	**Dimension 1**	**Dimension 2**	**Dimension 3**	**Dimension 4**	**Total**
	Desk review<br>20 interviews	20 interviews	20 interviews	Desk review<br>10 interviews	2 desk reviews<br>70 interviews
**HONDURAS**	**Dimension 1**	**Dimension 2**	**Dimension 3**	**Dimension 4**	**Total**
	Desk review<br>20 interviews	20 interviews	20 interviews	Desk review<br>10 interviews	2 desk reviews<br>70 interviews
**MÉXICO**	**Dimension 1**	**Dimension 2**	**Dimension 3**	**Dimension 4**	**Total**
	Desk review<br>20 interviews	20 interviews	20 interviews	Desk review<br>10 interviews	2 desk reviews<br>70 interviews
**URUGUAY**	**Dimension 1**	**Dimension 2**	**Dimension 3**	**Dimension 4**	**Total**
	Desk review<br>20 interviews	20 interviews	20 interviews	Desk review<br>10 interviews	2 desk reviews<br>70 interviews
**TOTAL 5 countries**	**Dimension 1**	**Dimension 2**	**Dimension 3**	**Dimension 4**	**TOTAL**

For peer review only

	5 desk reviews<br>100 interviews	100 interviews	100 interviews	5 desk reviews<br>50 interviews	**10 desk reviews**<br>**350 interviews**

Analysis

Document review data analysis (each country study)

For dimension 1, data from the document review will be charted and narratively synthesised based on the explicit wording and content of the text, following the categories of the WHO Global Abortion Policies Database. (9, 16). This WHO database contains information related to authorization and service-delivery requirements, conscientious objection, penalties, national SRH indicators, and UN Treaty Monitoring Body concluding observations among other documents.

The written words of the documents, however, take their meaning from their purpose and context, and often require interpretation. Therefore, for the interpretation of competing or inconsistent legal primary sources, legal secondary sources will be used. Additionally, a preliminary classification as barrier or facilitator for each topic will be carried out for the purpose to assess them in the interviews.

For dimension 4, data from the document review will be synthesised and indicators for abortion care will be gathered, classified, and analysed according to the AAAQ domains. The data extraction matrix will also be based on indicators for abortion care as identified as part of the WHO 2022 indicators for abortion, the Danish Institute for Human Rights reports on sexual and reproductive health and rights and the systematic review by Dennis *et.al.* (20-23) Gaps between the extraction matrix and the available indicators will be identified.

Interview data analysis (country studies)

Data from the interviews for each dimension will be summarized using an inductive thematic coding and a matrix organized around central aspects of the corresponding dimension. A content analysis will follow the coding to identify emerged themes and patterns among them. (24) Barriers and facilitators of policy implementation will be

For peer review only

identified in relation to the AAAQ framework in a data reduction matrix. The first step will be to extract the data from the interviews and place it within the topics of the data extraction matrix. Then, a second reading of the data in the matrix will take place, guided by specific questions for each dimension. Finally, the analysis of barriers, facilitators and strategies will be organized under the AAAQ matrix.

Interviews will also allow conducting a stakeholder analysis to map the actors who have had an interest and have affected or influenced the implementation of abortion policy (dimension 2), to better understand their role in the continuum supporters-blockers, following the questions suggested by Schmeer (25):

- Who are the most important abortion stakeholders (from a power and leadership perspective)?
- What are the different stakeholders' positions on abortion policies?
- What do the different stakeholders see as possible advantages or disadvantages of abortion policies?
- Which stakeholders form alliances? Is there potential for new alliances to form?

Identification and synthesis of barriers, facilitators, and strategies of comprehensive abortion policy implementation *(country studies)*

Once findings from dimensions 1 to 4 are gathered and processed, barriers, facilitators, and strategies to deal with them will be summarized into a data grid organised with the AAAQ framework. This aims to group the information from the four dimensions in one single document, which will shed light on strategies used towards comprehensive abortion policy implementation. (Fig.1)

After that, the information will be analysed using substantive themes (or codes or clusters of codes). The rearrangements and recombination may be facilitated by depicting the data graphically or by arranging them in lists, for example.

Lessons learned from the five country studies (regional synthesis)

At this final stage of the study, a regional synthesis exercise will be conducted to draw lessons on barriers, facilitators and the strategies provided by the five country studies. Once the first version of this synthesis exercise is ready, a validation process involving

For peer review only

members of all five national teams will take place to reach consensus on the synthesis. For this validation, an adjusted nominal group technique will be applied. (26)

The study process is summarized in Table 2:

Table 2 Study process

COUNTRIES	DATA COLLECTION	DATA ANALYSIS	FINAL PRODUCT
**ARGENTINA**	**D1-D4**<br>2 desk reviews<br>70 interviews	**D1-D4**<br>Barriers and facilitators<br>**Model AAAQ**	**1 country report**
**COLOMBIA**	**D1-D4**<br>2 desk reviews<br>70 interviews	**D1-D4**<br>Barriers and facilitators<br>**Model AAAQ**	**1 country report**
**HONDURAS**	**D1-D4**<br>2 desk reviews<br>70 interviews	**D1-D4**<br>Barriers and facilitators<br>**Model AAAQ**	**1 country report**
**MÉXICO**	**D1-D4**<br>2 desk reviews<br>70 interviews	**D1-D4**<br>Barriers and facilitators<br>**Model AAAQ**	**1 country report**
**URUGUAY**	**D1-D4**<br>2 desk reviews<br>70 interviews	**D1-D4**<br>Barriers and facilitators<br>**Model AAAQ**	**1 country report**
**TOTAL 5 countries**	**10 desk reviews and 350 interviews**	Barriers and facilitators per dimension in the 5 countries<br>**Model AAAQ**	**5 country reports**
**REGIONAL LEVEL**		**Synthesis exercise of the 5 country reports**	**1 regional report on lessons learnt**

Ethical considerations

The project has been approved by the World Health Organisation Research Ethics Review Committee. Moreover, it has been approved by the local Research Ethics Committee in the five participating countries: Centro Rosarino de Estudios Perinatales (Argentina), Profamilia CEIP (Colombia), Universidad Autónoma de Honduras CEIB (Honduras), Instituto Nacional de Salud Pública (México) and Centro Hospitalario Pereira Rossell (Uruguay).

The study will respect the ethical safeguards necessary to obtain informed and voluntary consent and preserve the anonymity of the key informants interviewed. Names of

For peer review only

individuals and organizations will not be used in any final or publicly available product resulting from this research. Interviewers will be professionals with proven experience to guarantee ethical principles in conducting the interviews, date and time will be based on the participant's availability.

Data collected in this study will be managed, stored, analysed, and results will be disseminated with careful attention to protecting human subjects' rights to privacy and confidentiality. Specifically, data will be treated in compliance with and approved by the WHO's Research Ethics Review Committee (ERC) and Centro Rosarino de Estudios Perinatales Independent Ethics Committee.

Dissemination

Findings from the study will be disseminated through a multipurpose strategy to target diverse audiences so as to foster the use of the study findings to inform the public debate agenda and policy implementation at national level. The dissemination strategy will include:

-Protocol publication: the protocol will be submitted to a peer reviewed journal to disseminate the design and methodology of the multi-country study.

-Peer-reviewed publication: Academic article(s) will be submitted to an international journal to disseminate main findings of the comparative analysis of barriers and facilitators of quality abortion policy implementation in the selected countries.

-National dissemination workshop: this workshop will gather diverse political, academic and policy actors to discuss main findings and discuss barriers and facilitators of quality abortion policy implementation.

-Regional dissemination workshop: this workshop will gather diverse political, academic and policy actors to discuss main findings of the comparative analysis of barriers and facilitators of quality abortion policy implementation in the Latin American region.

Strengths and limitations

A strength of this study is the assessment of different dimensions and actors of abortion policy implementation, which should provide a comprehensive and more precise

understanding of the factors that influence the implementation of these types of health policies. Nevertheless, from all complex social, legal, cultural, and ethical factors shaping abortion care and policies, this research will focus on four to identify relevant barriers and facilitators to abortion policies, using the AAAQ framework. In this process, women's voices are not included directly, which could be a limitation. However, women's organizations, which usually have relevant and updated information about women's experiences with abortion, are participants in this study as stakeholders.

Another strength of this project is that, as a multi-country study, it encompasses five countries from restrictive to more liberalised environments. This will show different contexts while looking at common patterns regarding barriers, facilitators, and strategies. This data will be collected through standardized instruments and validated by the teams involved in the project. However, there are challenges in providing a systematic comparison of heterogeneous countries. Moreover, the samples in this study may not reflect the heterogeneity of institutions and the proportionality of the population. To capture this complex scenario in each of the five country-case studies, local interview guides will be adjusted and expanded based on contextual factors.

This study will focus on factors influencing implementation and will not analyse how these factors might correspond to other aspects, such as the evaluation of abortion policies, the estimation of satisfaction with the care provided or the magnitude and severity of abortion-related complications in relation to policy implementation. Future research may consider including these and other aspects.

Finally, principal and country investigators will commit to include junior researchers in the development of the study. By conducting this research project, the coordinating team and the local teams will strengthen their skills, including those of junior researchers, to provide more responsive and sustainable policies that address women's needs and rights.

References

1. World Health Organization (WHO). Abortion care guideline. 2022. Available at: https://www.who.int/publications/i/item/9789240039483
2. World Health Organization (WHO). WHO Unsafe abortion: global and regional estimates of incidence of unsafe abortion and associated mortality in 2008. 2011.

http://apps.who.int/iris/bitstream/handle/10665/44529/9789241501118_eng.pdf;jsessionid=6E4DB04A17643FA2E25D8EC75C51E609?sequence=1

3. Dawson A, Bateson D, Estoesta J, Sullivan E. Towards comprehensive early abortion service delivery in high income countries: insights for improving universal access to abortion in Australia. BMC Health Serv Res. 2016; 16, 612.
4. Abortion Worldwide: 20 Years of Reform. Center for Reproductive Rights. 2014. https://www.reproductiverights.org/sites/crr.civicactions.net/files/documents/20Years_Reform_Report.pdf
5. International Human Rights Law and Abortion in Latin America. Human Rights and Abortion. Human Rights Watch. 2005. https://www.hrw.org/legacy/backgrounder/wrd/wrd0106/wrd0106.pdf
6. Abortion in Latin America and the Caribbean. Fact sheet. Guttmacher Institute. 2018. https://www.guttmacher.org/sites/default/files/factsheet/fb_iaw.pdf
7. Doran F, Nancarrow S. Barriers and facilitators of access to first-trimester abortion services for women in the developed world: a systematic review. Journal of Family Planning and Reproductive Health Care. 2015; 41:170-180.
8. Abortion and Reproductive Rights in Latin America: Implications for democracy. Inter-American Dialogue and the Centre for Reproductive Rights. 2015. https://www.thedialogue.org/wp-content/uploads/2019/07/AbortionandReproductiveRightsinLatinAmerica.pdf
9. Johnson BR Jr, Lavelanet AF, Schlitt S. Global Abortion Policies Database: a new approach to strengthening knowledge on laws, policies, and human rights standards. BMC Int Health Hum Rights. 2018;12,18(1),35.
10. Dawson A, Bateson D, Estoesta J, Sullivan E. Towards comprehensive early abortion service delivery in high income countries: insights for improving universal access to abortion in Australia. BMC Health Serv Res. 2016; 16, 612.

For peer review only

11. Rowlands S, Lopez Arregui E. How health services can improve access to abortion. The European Journal of Contraception and Reproductive Health Care. 2016; 21, 1, 1–3
12. Mainey L, O'Mullan C, Reid-Searl K, Taylor A, Baird K. The role of nurses and midwives in the provision of abortion care: A scoping review. Journal of Clinical Nursing. 2020; 29(9-10), 1513–1526.
13. Olavarrieta C, Ganatra B, Sorhaindo A, Karver T, Seuc, A. et al. Nurse versus physician-provision of early medical abortion in Mexico: a randomized controlled non-inferiority trial. Bulletin of the World Health Organization. 2015; 93 (4), 249-258.
14. Ramón Michel A, Romero M, Ramos S, Gonzalez Velez C. Seguimiento de la CIPD en América Latina y el Caribe después de 2014. 2014; DOI 10.13140/RG.2.2.24107.54562.
15. World Health Organization (WHO). Gender, equity and human rights. 2016. https://www.who.int/gender-equity-rights/knowledge/AAAQ.pdf
16. Erdman JN, Johnson BR Jr. Access to knowledge and the Global Abortion Policies Database. Int J Gynaecol Obstet. 2018 ;142(1):120-124.
17. Ramón Michel A. Barreras en el acceso a los abortos legales: una mirada a las regulaciones sanitarias que incluyen el uso del misoprostol. 2013. https://clacaidigital.info/bitstream/handle/123456789/489/Misoprostol.reg.barreras.pdf?sequence=5&isAllowed=y
18. Adams WC. Conducting Semi-Structured Interviews. In: Wholey JS, Harty HP, Newcomer KE, editors. Handbook of Practical Program Evaluation. San Francisco: Jossey - Bass; 2015, p. 492–505.
19. Yin RK. Qualitative research from start to finish. The Guilford Press. 2011.
20. World Health Organization (WHO). Safe Abortion: Technical and Policy Guidance for Health Systems. 2012. https://www.who.int/reproductivehealth/publications/unsafe_abortion/9789241548434/en
21. World Health Organization (WHO). Sexual health, human rights and the law. 2015.

For peer review only

https://apps.who.int/iris/bitstream/handle/10665/175556/9789241564984_eng.pd?sequence=1

22. Kähler L, Villumsen M, Holst Jensen M, Falk Paarup P. AAAQ & Sexual and Reproductive Health and Rights. International Indicators for Availability, Accessibility, Acceptability and Quality. The Danish Institute for Human Rights. 2017. https://www.humanrights.dk/sites/humanrights.dk/files/media/migrated/aaaq-srhr_issue_paper_dihr_2017_english.pdf
23. Dennis A, Blanchard K, Bessenaar T. Identifying indicators for quality abortion care: a systematic literature review. J Fam Plann Reprod Health Care. 2017;43(1):7-15.
24. Vaismoradi, M., Turunen, H. and Bondas, T. Qualitative descriptive study. Nurs Health Sci. 2013, 15: 398-405.
25. Schmeer, K. Policy Toolkit for Strengthening Health Sector Reform. Stakeholder Analysis Guidelines. Chapter 2. Stakeholder analysis at a glance. LAC HSR. 2000.
26. Manera K, Hanson C, Gutman T, Tong A. Consensus Methods: Nominal Group Technique. In: Liamputtong P, editor. Handbook of Research Methods in Health Social Sciences. Singapore: Springer Nature; 2019, 737-750.

Authors' contributions

AL, CG, ARM, SR, GC, MR contributed to the conception and design of the study protocol, which was discussed with the rest of the authors. CG, ARM, SR, GC, MR, BC, RGPL and AL agreed on the methodological aspects and participated in developing the protocol, reviewing the draft, and making substantial revisions. CG wrote the original outline. ARM together with CG were responsible of the writing, reviewing, and editing the subsequent versions of the protocol. ARM oversaw developing dimension 1 section. SR was in charge of developing dimension 2. MR was in charge of developing dimension 3. GC and CG were in charge of dimension 4. BC developed and wrote all ethics considerations and prepared the original figures with SR and CG. BC and MVO revised it for intellectual content and provided feedback. CG and ARM led the manuscript writing. CG and ARM, with the help of MVO, coordinated the steps for article development and wrote the draft of the article together. AL, SR, GC, MR and

For peer review only

RGPL contributed to manuscript writing and revision. All authors contributed to read and approved the submitted version.

Funding statement

This project is funded by the UNDP-UNFPA-UNICEF-WHO-World Bank Special Programme of Research, Development and Research Training in Human Reproduction (HRP), Department of Sexual and Reproductive Health and Research, World Health Organization, 20 Avenue Appia, 1211 Geneva, Switzerland. The content of this article is solely the responsibility of the authors and does not reflect the views of the referred programme.

Competing interests

The authors declare that they have no competing interests.

Acknowledgements

We thank the country principal investigators for their comments to the protocol and contributions regarding country specific information: Edgardo Abalos (Argentina), Rocío Murad (Colombia), Jakeline Álger (Honduras), Karla Berdichevsky (México), Karla Flores (México) y Cecilia Stapff (Uruguay). We also thank Agostina Allori for reviewing dimension 1 and Daniel Giordano for his inputs regarding data management and data security aspects.

Authors' details

1. Centro Rosarino de Estudios Perinatales (CREP), Rosario, Argentina.

2. Centro de Estudios de Estado y Sociedad (CEDES), Buenos Aires, Argentina.

3. Consejo Nacional de Investigaciones Científicas y Tecnológicas (CONICET), Argentina.

4. Centro Latinoamericano de Perinatología/Salud de la Mujer y Reproductiva (CLAP/SMR) of the Pan American Health Organization (PAHO).

5. Department of Reproductive Health and Research, World Health Organization (WHO), Geneva, Switzerland.

For peer review only

Fig.1. Research framework: dimensions & methods

For peer review only

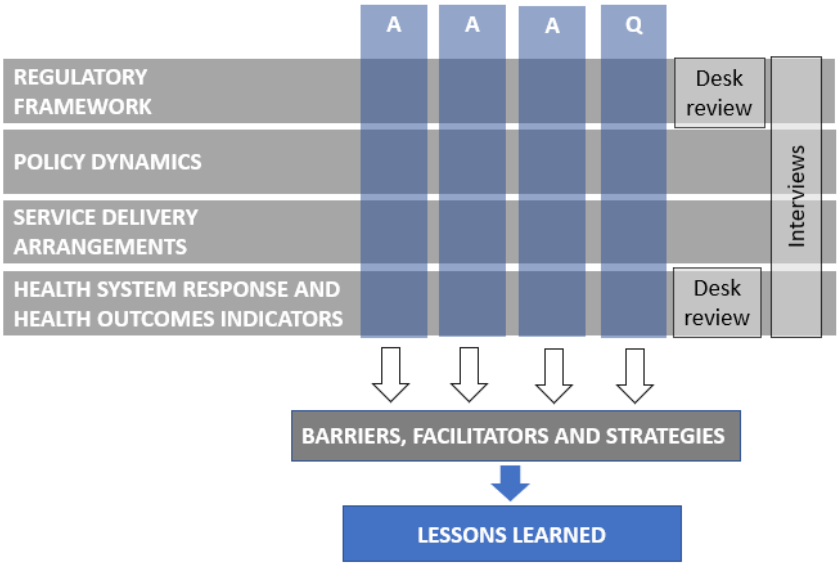

peer review only

Multi-country research on comprehensive abortion policy implementation in Latin America: a mixed-methods study protocol

Journal:	*BMJ Open*
Manuscript ID	bmjopen-2023-073617.R1
Article Type:	Protocol
Date Submitted by the Author:	14-Oct-2023

Complete List of Authors:	Gialdini, Celina; Centro Rosarino de Estudios Perinatales; Blanquerna Ramon Llull University Faculty of Health Sciences<br>Ramón Michel, Agustina; CEDES<br>Romero, Mariana; CEDES; CONICET<br>Ramos, Silvina; CEDES<br>Carroli, Guillermo; CREP<br>Carroli, Berenise; CREP<br>Gomez Ponce de León, Rodolfo; Pan American Health Organization, CLAP WRH<br>Vila Ortiz, Mercedes; CREP<br>Lavelanet, Antonella ; WHO, ; World Health Organization

**Primary Subject Heading**:	Public health
Secondary Subject Heading:	Health policy, Sexual health, Obstetrics and gynaecology
Keywords:	PUBLIC HEALTH, Health policy < HEALTH SERVICES ADMINISTRATION & MANAGEMENT, SEXUAL MEDICINE, Health Services Accessibility, Organisation of health services < HEALTH SERVICES ADMINISTRATION & MANAGEMENT, Quality in health care < HEALTH SERVICES ADMINISTRATION & MANAGEMENT

SCHOLARONE™
Manuscripts

Multi-country research on comprehensive abortion policy implementation in Latin America: a mixed-methods study protocol

Authors: Gialdini, C.*[1,2], Ramón Michel, A.[3], Romero, M.[3,4], Ramos, S.[3], Carroli, G.[1], Carroli, B. [1], Gomez Ponce de León, R.[5], Vila Ortiz, M. [1], Lavelanet, A[6].

[1] Centro Rosarino de Estudios Perinatales (CREP)
[2] Blanquerna Faculty of Health Sciences, Ramon Llull University
[3] Centro de Estudios de Estado y Sociedad (CEDES)
[4] Consejo Nacional de Investigaciones Científicas y Técnicas (CONICET)
[5] Pan American Health Organization, CLAP WRH (PAHO)
[6] Wordl Helath Organization (WHO)

*Corresponding author, cgialdini@crep.org.ar, Centro Rosarino de Estudios Perinatales

Funder: UNDP-UNFPA-UNICEF-WHO-World Bank Special Programme of Research, Development and Research Training in Human Reproduction (HRP), Department of Sexual and Reproductive Health and Research, World Health Organization (Study number A66023)

Abstract

Introduction

Access to comprehensive abortion care could prevent the death of between 13,865 and 38,940 women and the associated morbidity of five million women worldwide. There have been some important improvements in Latin America in terms of laws and policies on abortion. However, the predominant environment is still restricted and many women, adolescents, and girls still face multiple barriers to exercise their reproductive rights. This research will systematically assess comprehensive abortion policies in five Latin American countries (Argentina, Colombia, Honduras, Mexico, and Uruguay). The aim is to identify barriers, facilitators, and strategies to the implementation of abortion policies, looking at four key dimensions −regulatory framework, abortion policy dynamics, abortion service delivery, and health system and health outcomes indicators− to draw cross-cutting lessons learned to improve current implementation and inform future safe abortion policy development.

Methods and analysis

A mixed-method design will be used in the five countries to address the four dimensions through the availability, accessibility, acceptability, and quality of care model. The data collection tools include desk reviews and semi-structured interviews with key actors. Analysis will be performed using thematic analysis and stakeholder analysis. A regional synthesis exercise will be conducted to draw lessons on barriers, facilitators, and the strategies to deal with them.

Ethical considerations and dissemination

The project has been approved by the World Health Organisation Research Ethics Review Committee (ID: A66023) and by the local Research Ethics Committees.

Informed consent will be obtained. Data will be treated with careful attention to protecting privacy and confidentiality.

Findings from the study will be disseminated through a multipurpose strategy to target diverse audiences to foster the use of the study findings to inform the public debate agenda and policy implementation at national level. The strategy will include academic, advocacy and policy arenas and actors: peer-reviewed publication, national and regional dissemination workshops.

Strengths and limitations

- The study encompasses five countries with a variety of legal environments regarding abortion in order to show different contexts while looking at common patterns regarding barriers, facilitators, and strategies.
- From all complex social, legal, cultural, and ethical factors shaping abortion care and policies, this research will focus on four, using the AAAQ framework.
- This study will focus on factors influencing implementation.
- Data will be collected through standardized instruments, which might not reflect the heterogeneity among and within participating countries.

Introduction

Access to comprehensive evidence-based abortion care could prevent the death of between 13,865 and 38,940 women (1) and the associated morbidity of five million women worldwide. (2) Improving access to quality abortion care is an essential strategy for the provision of universal access to reproductive health and for the achievement of the United Nations Sustainable Development Goals (SDG): good health and well-being

For peer review only - http://bmjopen.bmj.com/site/about/guidelines.xhtml

and gender inequality (SDGs 3 and 5). Women's access to sexual and reproductive rights can also contribute to reproductive empowerment, and therefore address additional SDGs such as quality education (SDG 4), sanitation and hygiene (SDG 6), decent work and economic growth (SDG 8), reduce inequalities (SDG 10) and develop peace, justice, and strong institutions (SDG 16). (3)

Since the adoption of the Programme of Action (PoA) of the International Conference on Population and Development (ICPD) 28 years ago, countries have been called to strengthen their commitment to women's health by addressing unsafe abortion and supporting a woman's right to decide. (4) Although some improvements in terms of policies on sexual and reproductive health −including law reforms− have been achieved, many women in Latin America face multiple barriers to exercise their reproductive rights. (5)

Obstacles to quality abortion access are a matter of human rights and of public health: in this region, at least 10% of all maternal deaths are a consequence of unsafe abortions and near 760,000 women are hospitalized per year due to unsafe abortion related complications, which violates their rights to life and health. (6) The obstacles faced by women, adolescents and girls when trying to access quality abortion include the interpretation and the implementation of the grounds (i.e., legal indications under which is legal to have an abortion) for having a legal abortion, the lack of access to information, stigma, restricted type of health-care providers and scarcity of facilities that can lawfully provide services, among others. (7) These barriers can disproportionately affect specific groups, such as adolescents, women that are poor, indigenous women, women living in small towns, and other women in vulnerable social conditions.

In this context, abortion laws have been difficult to reform. Most Latin American countries have restrictive abortion laws, allowing legal termination of pregnancy only under certain grounds such as rape, severe fetal impairment or when the woman's life or health is at risk. (8) Abortion is legal without restriction as to reason in the first trimester of pregnancy in Argentina, Cuba, Uruguay, and nine states of Mexico (Mexico City, Oaxaca, Hidalgo, Veracruz, Baja California, Baja California Sur, Sinaola, Guerrero and Colima). Moreover, in February 2022, the Colombian

For peer review only

Constitutional Court ruled that abortion should be decriminalized up to 24 weeks of pregnancy.

Nevertheless, some countries have been able to improve availability, accessibility, acceptability and quality of abortion services and care by resorting to other strategies. Court judgments and public health guidelines have been used to expand the interpretation of the circumstances under which abortion is allowed. In settings with very restrictive legal environments, like Honduras, guidelines to manage post-abortion care have also been issued. (9)

Taking this regional context into consideration, this research will systematically look at comprehensive abortion policies in five Latin American countries −Argentina, Colombia, Honduras, Mexico, and Uruguay− to understand how context, content, actors and processes affect the implementation of comprehensive abortion policies. The aim is to assess abortion regulations[1] and their use in practice, the policy dynamics, the service delivery arrangements, and the abortion-related health indicators and how they are monitored and evaluated, to identify barriers and facilitators to the implementation of comprehensive abortion policy and the strategies used to address them in each country. Finally, this study will identify similarities and differences among barriers, facilitators, and strategies of policy implementation across countries, and draw lessons learned from these implementation processes and the strategies to improve access to evidence-based abortion care.

Methods and analysis

Design and framework

This research study will look at the context, content, actors, and processes affecting the implementation and monitoring of comprehensive abortion policies and comprises five country-case studies and a regional synthesis of findings. A mixed-method design using diverse data gathering techniques and analysis strategies will assess four key dimensions of abortion policy[2]:

[1] By Regulations we mean rules or guidelines established and enforced by an authority such as constitutional clauses, laws, decrees, and other sort of administrative regulations

For peer review only - http://bmjopen.bmj.com/site/about/guidelines.xhtml

- The **regulatory framework** (dimension 1)
- The **policy dynamics** (dimension 2)
- The **service delivery arrangements** (dimension 3)
- The **health system and health outcomes indicators** (dimension 4)

Dimension 1 (regulatory framework) will assess how and to what extent regulations −both written and in practice− pose as barriers and facilitators to quality abortion access, and the strategies to deal with them. It will address the traits and use of existing abortion related regulations. This dimension encompasses laws, resolutions, decrees, health standards and health guidelines, technical guidelines, and other official documents with or without legal status. First, a descriptive analysis of the regulatory framework will be performed, and then an examination of the practice.

Dimension 2 (policy dynamics) will assess how aspects such as agenda setting, design and implementation processes, and governance and actors' involvement, pose as barriers and facilitators of abortion policy, and how they are addressed through different strategies. Agenda setting will be assessed looking at the political and social conditions that place abortion policy on the public agenda. Design and implementation aspects will focus on the shaping of content in policy formulation and on the main challenges of implementation. Governance issues such as the capacity of health authorities to rule, reinforce and have control of the implementation of abortion policy for the provision and access to comprehensive abortion care will be assessed. The policy dynamics will be addressed from the perspective of policy makers, health services managers and women's organizations.

Dimension 3 (service delivery arrangements) will assess how health services are organised to ensure access to quality abortion care, and how barriers and facilitators are addressed by different strategies. (10-14)

Dimension 4 (health system and health outcomes indicators) will assess what information on health system and health outcomes indicators related to abortion care are monitored and evaluated. It will address the process of generating, collecting, reporting, monitoring,

---

[2] By Policy we mean a system of laws, regulatory measures, courses of action, and funding priorities concerning a given topic promulgated, in this case, abortion.

For peer review only

and evaluating health system and health outcomes indicators, and how this evaluation accounts for policy implementation progress and quality of care oversight.

We will assess these dimensions through availability, accessibility, acceptability, and quality of care as framed in the **AAAQ model**. This framework will be used to gather and systematize the data coming from the two data collection instruments: semi-structured interviews and document review. *Availability* refers to the need of having enough functioning public health and health care facilities, goods, and services. *Accessibility* refers to the need of making all those facilities, goods, and services physically accessible and affordable to everyone within the jurisdiction of the State party without discrimination. *Acceptability* refers to the need of the health system responses to be respectful of the law, medical ethics, and to be culturally appropriate and sensitive to gender and age. *Quality of care* also refers to the need for health goods and services to be scientifically and medically approved and of good quality following WHO standards. (15) Specifically, *quality of abortion care* refers to the fact that the provision of abortion services should be effective, efficient, accessible, acceptable (patient centred), equitable and safe. (1)

As a result of the appraisal of the four dimensions, we will identify the **barriers and facilitators** to availability, accessibility, acceptability, and quality of care regarding abortion related to abortion policy implementation.

Finally, we will synthesize findings from the case studies to draw lessons learned on cross-cutting barriers and facilitators, as well as the similarities and differences on how comprehensive abortion policies are implemented in the participant countries. These lessons learned can be used to inform future decision-making across the region and other sociocultural and geopolitical contexts. (See Fig. 1).

For this study, comprehensive abortion care includes the elements of induced abortion under legal indications as well as all the elements of post abortion care: information, counselling, treatment, contraceptive services, reproductive health services, community, and service providers' partnerships.

Fig.1. Research framework: dimensions & methods

For peer review only - http://bmjopen.bmj.com/site/about/guidelines.xhtml

<u>Settings</u>

We will conduct case-studies in five selected countries: Argentina, Colombia, Honduras, Mexico, and Uruguay. The selection of settings addresses diversity in abortion regulations as well as diversity in countries' institutional political organization across a continuum of abortion related legal access. We aim to include countries going from an absolute legal restrictive environment with only post-abortion care provision to countries with less restrictive legal status of abortion, availability of abortion guidelines, or registration and mifepristone and misoprostol availability, two medicines used in the medical abortion process. The selection also considers common issues on abortion policy implementation and indicators among countries. Country profiles are summarized in Table 1.

Table 1: Country profile

COUNTRIES / DIMENSIONS	Argentina	Colombia	Honduras	México[3]	Uruguay
**Socio economic profile**	- 42% of the population lives under poverty[4]<br>- 42, 3 Gini Coefficient[5]<br>- High level of Inflation. The second country with highest (triple digit inflation)<br>- A historically large middle class is shrinking	- 42% of the population lives under poverty[6]<br>-54, 2 Gini Coefficient[7]<br>- Under a peace agreement process, with still sites with high levels of violence and isolation	- 48% of the population lives under poverty[8]<br>- 48, 2 Gini Coefficient[9]<br>- The size of the middle class was among the smallest in the region (18% compared to an average 41%)	- 44% of the population lives under poverty[10]<br>- 45, 4 Gini Coefficient[11]<br>- High levels of social and political violence connected with the illegal drug market	- 11% of the population lives under poverty[12]<br>- 40, 3 Gini Coefficient[13]<br>- Its middle class is the largest in the Americas, representing more than 60% of its population[14]

---

[3] Mexico has different regulation it each state, so the references here are regarding the national level

[4] World Bank, 'Pobreza en América Latina', *Statista*, 2022, https://es.statista.com/temas/8701/pobreza-en-america-latina/.

[5] World Bank, 'Países con mayor desigualdad en el coeficiente Gini en América Latina', *Statista*, 2021, https://es.statista.com/estadisticas/1267584/latinoamerica-coeficiente-gini-desigualdad-de-ingresos-por-pais/..

[6] World Bank, 'Pobreza en América Latina', op. cit. 2.

[7] World Bank, 'Países con mayor desigualdad en el coeficiente Gini en América Latina', op. cit. 3.

[8] World Bank, 'Pobreza en América Latina', op. cit. 2.

[9] World Bank, 'Países con mayor desigualdad en el coeficiente Gini en América Latina', op. cit. 3.

[10] World Bank, 'Pobreza en América Latina', op. cit. 2.

[11] World Bank, 'Países con mayor desigualdad en el coeficiente Gini en América Latina', op. cit. 3.

[12] World Bank, 'Pobreza en América Latina', op. cit. 2.

[13] World Bank, 'Países con mayor desigualdad en el coeficiente Gini en América Latina', op. cit. 3.

For peer review only

			- High levels of violence: over 38 homicides per 100,000 inhabitants		
**Division of the geographical power**	Federalism with shared competences between national and local government	Centralist with local powers	Unitarian	Strong Federalism	Centralist
**Abortion legal model**	Mixed (indications and voluntary interruption of pregnancy) with partial decriminalization	Mixed with total decriminalized up to 24 weeks	Total ban	Mixed at some states<br><br>Indication model in other states.	Mixed with partial decriminalization
**Main characteristics of abortion regulation**	-The law allows any health professional to provide abortion services<br>-No rape report is required for access legal abortion under the rape indication<br>-There is no abortion committee<br>-Conscientious objection is allowed but with limitations and only at an individual level	-The law allows any health professional to provide abortion services<br>-There is no abortion committee<br>-Ambulatory abortion is accepted<br>-No rape report is required for access legal abortion under the rape indication<br>-Conscientious objection is allowed but with limitations and only at an individual level	----------	-In some states rape report is required<br>-There is no abortion committee<br>-Conscientious objection is allowed but with limitations and only at an individual level at the national level but there is still	-There is an abortion committee<br>-Medical based regulation, with several other requirements<br>-Conscientious objection is allowed in broad terms, both individual and institutional
**Protocol of safe and legal abortion**	Yes<br>National	Yes<br>National	No<br>Just Protocol for Management of Obstetric Complication	Yes<br>Locals with variations	Yes<br>National
**Institutional adjudication**	Congress	Court	----------	Local legislatures	Congress
**Level where care is provided**	First and second level	First and second level	Second level	First and second level	Second level
**Health service arrangements**	Public health sector is the main abortion provider together with the community sector (also called acompañantes or informal health system)	Private clinics (Pro Familia and Orientame) are two of the main abortion providers	Some Public hospital are the ones proving abortion care	Public specialized abortion clinics are the main providers in México City	Public health sector is the main provider, and private clinics are not promoted

<u>Data collection and tools</u>

We will utilize two data collection methods: desk review (for dimensions 1 and 4) and semi-structured interviews (for dimensions 1, 2, 3, and 4). The data collection process is summarized in Table 2. The quantitative data will not only inform the qualitative process, but will be integrated into the analysis to better elucidate different aspects of the dimensions, thereby allowing for a robust process of triangulation.

Desk review

[14] World Bank, 'Uruguay: panorama general', *World Bank*, 2023, https://www.bancomundial.org/es/country/uruguay/overview.

For peer review only - http://bmjopen.bmj.com/site/about/guidelines.xhtml

For dimension 1, we will review the texts of regulations to identify and categorize abortion related regulations, we will structure a data extraction matrix into two domains:

− the abortion regulatory framework, which refers to the specific regulations and practices related to abortion.

− the legal environment, which includes regulations that are not specifically about abortion but have a direct impact on access to abortion health services (e.g., laws regarding patient confidentiality, norms regarding minors' consent, norms on mental or intellectual disabilities, laws related to sexual violence).

For dimension 4, we will conduct a systematic search for country-specific statistics, reports and documents. National official reports and databases containing processes, outputs, or outcome indicators relevant to abortion care at national level will be reviewed and gaps in indicator reporting, monitoring and evaluation will be identified.

We will base the data extraction matrix for dimension 1, on the topics included in the WHO Global Abortion Policies Database (9, 16) and in the report "*Barreras en el acceso a los abortos legales: una mirada a las regulaciones sanitarias que incluyen el uso del misoprostol*". (17) For dimension 4, a data extraction matrix structured with the AAAQ domains will serve to identify and categorize abortion related indicators. From each reported indicator, we will collect data on indicator definition, method of measurement, data collection methodology and frequency, date of publication, source of information and indicator value. If data are stratified (e.g., race, gender, age, urban/rural), this information will also be collected.

Semi-structured interviews

Semi-structured interviews (18) will explore:

- how legal and regulatory instruments are used, how they pose as barriers and facilitators to abortion service provision and access, and how regulations are translated into practice (dimension 1).
- the policy dynamics related to agenda setting, governance issues, financing schemes, and actors' involvement (dimension 2).
- which are the service arrangements adopted according to the different settings, human resources, and the technologies available, and which were the triggers for the model/different models currently used (dimension 3); and

For peer review only

- which approaches each country uses for monitoring abortion-related care and for the oversight of maternal mortality, near miss and quality of care and how monitoring and evaluation of process –and outcomes– indicators related to abortion are operationalized (dimension 4).

Interviews will be conducted by an experienced interviewer with social science or public health background with legal or programmatic knowledge. The interviews will be recorded after informed consent has been obtained and then transcribed. The interviews may be conducted in person or through digital technology.

<u>Participants</u>

For the interviews, we will use a purposive sample of twenty key informants for dimensions 1 to 3 and 10 key informants for dimension 4 in each country. This sample seeks to obtain a broad range of perspectives on the subject (19). The selected subjects will be key informants in the abortion policy domain, encompassing different categories: health providers, sexual and reproductive health policy decision makers, researchers, leaders of women's organizations, judicial agents, politicians, opinion leaders, etc.

The sample size is a target overall number, which will be adjusted during the data collection process. Twenty key informants for dimension 1 to 3 and ten key informants for dimension 4 in each country is considered a feasible sample size that should allow for variability along the different categories (see Table 1).

The identification of participants is expected to be different depending on how broad or narrow the categories or levels from where they are identified are. The criteria of variability of forums or levels aims at obtaining diversity and having those that yield the most plentiful data in the matter.

We will identify the key informants through a mapping of actors. A first round of mapping will be based on teams' expertise and networks. Narrow-level participants such as decision makers (who are few in each national abortion domain) are expected to be easily identified. For broad categories, such as health providers and activists (who are many and disperse in different settings), the selection strategy will be ultimately defined with each country's principal investigator to achieve a convenience sample that accounts for

For peer review only - http://bmjopen.bmj.com/site/about/guidelines.xhtml

282 variability. There may be a second round of mapping to select knowledgeable
283 stakeholders identified by the interviewees from the first round.
284
285 Table 2 Data collection
286

**ARGENTINA**	**Dimension 1**	**Dimension 2**	**Dimension 3**	**Dimension 4**	**Total**
	Desk review<br>20 interviews	20 interviews	20 interviews	Desk review<br>10 interviews	2 desk reviews<br>70 interviews
**COLOMBIA**	**Dimension 1**	**Dimension 2**	**Dimension 3**	**Dimension 4**	**Total**
	Desk review<br>20 interviews	20 interviews	20 interviews	Desk review<br>10 interviews	2 desk reviews<br>70 interviews
**HONDURAS**	**Dimension 1**	**Dimension 2**	**Dimension 3**	**Dimension 4**	**Total**
	Desk review<br>20 interviews	20 interviews	20 interviews	Desk review<br>10 interviews	2 desk reviews<br>70 interviews
**MÉXICO**	**Dimension 1**	**Dimension 2**	**Dimension 3**	**Dimension 4**	**Total**
	Desk review<br>20 interviews	20 interviews	20 interviews	Desk review<br>10 interviews	2 desk reviews<br>70 interviews
**URUGUAY**	**Dimension 1**	**Dimension 2**	**Dimension 3**	**Dimension 4**	**Total**
	Desk review<br>20 interviews	20 interviews	20 interviews	Desk review<br>10 interviews	2 desk reviews<br>70 interviews
**TOTAL 5 countries**	**Dimension 1**	**Dimension 2**	**Dimension 3**	**Dimension 4**	**TOTAL**
	5 desk reviews<br>100 interviews	100 interviews	100 interviews	5 desk reviews<br>50 interviews	**10 desk reviews**<br>**350 interviews**

287
288 Analysis
289
290 *Document review data analysis (each country study)*
291
292 For dimension 1, we will chart and narratively synthesize data from the document review
293 based on the explicit wording and content of the text, following the categories of the
294 WHO Global Abortion Policies Database. (9, 16). This WHO database contains
295 information related to authorization and service-delivery requirements, conscientious
296 objection, penalties, national SRH indicators, and UN Treaty Monitoring Body
297 concluding observations among other documents.
298
299 The written words of the documents, however, take their meaning from their purpose and
300 context, and often require interpretation. Therefore, for the interpretation of competing or
301 inconsistent legal primary sources, legal secondary sources will be used. Additionally, a

For peer review only

preliminary classification as barrier or facilitator for each topic will be carried out for the purpose to assess them in the interviews.

For dimension 4, we will synthesize data from the document review and further collect indicators for abortion care; these will be classified and analysed according to the AAAQ domains. The data extraction matrix will also be based on indicators for abortion care as identified as part of the WHO 2022 indicators for abortion, the Danish Institute for Human Rights reports on sexual and reproductive health and rights and the systematic review by Dennis *et.al.* (20-23) Gaps between the extraction matrix and the available indicators will be identified.

Desk reviews will help researchers gain a comprehensive understanding of the subject matter and identify key themes or gaps in knowledge. Interviews will be informed by the desk review and collection of country specific statistics.

Interview data analysis (country studies)

We will use an inductive thematic coding approach with the data from the interviews for each dimension and organize a matrix round central aspects of the corresponding dimension. A content analysis will follow the coding to identify emerged themes and patterns among them. (24) Barriers and facilitators of policy implementation will be identified in relation to the AAAQ framework in a data reduction matrix. The first step will be to extract the data from the interviews and place it within the topics of the data extraction matrix. Then, a second reading of the data in the matrix will take place, guided by specific questions for each dimension. Finally, the analysis of barriers, facilitators and strategies will be organized under the AAAQ matrix.

Interviews will also allow conducting a stakeholder analysis to map the actors who have had an interest and have affected or influenced the implementation of abortion policy (dimension 2), to better understand their role in the continuum supporters-blockers, following the questions suggested by Schmeer (25):

- Who are the most important abortion stakeholders (from a power and leadership perspective)?
- What are the different stakeholders' positions on abortion policies?

For peer review only - http://bmjopen.bmj.com/site/about/guidelines.xhtml

- What do the different stakeholders see as possible advantages or disadvantages of abortion policies?
- Which stakeholders form alliances? Is there potential for new alliances to form?

Identification and synthesis of barriers, facilitators, and strategies of comprehensive abortion policy implementation *(country studies)*

We will then apply the findings from dimensions 1 to 4 to a data grid organised with the AAAQ framework to better illustrate the barriers, facilitators, and strategies to deal with them. This aims to group the information from the four dimensions in one single document, which will shed light on strategies used towards comprehensive abortion policy implementation. (Fig.1)

We will analyse this information using substantive themes (or codes or clusters of codes). The rearrangements and recombination may be facilitated by depicting the data graphically or by arranging them in lists, for example.

Lessons learned from the five country studies (regional synthesis)

At this final stage of the study, we will conduct a regional synthesis exercise to draw lessons on barriers, facilitators and the strategies provided by the five country studies. Once the first version of this synthesis exercise is ready, we will conduct a validation process involving members of all five national teams to reach consensus on the synthesis. For this validation, an adjusted nominal group technique will be applied. (26)

Summary of study process

This study is expected to start in February 2024 and finish in May 2025. The study process is summarized in Table 3:

Table 3 Study process

COUNTRIES	DATA COLLECTION	DATA ANALYSIS	FINAL PRODUCT
**ARGENTINA**	**D1-D4**<br>2 desk reviews<br>70 interviews	**D1-D4**<br>Barriers and facilitators<br>**Model AAAQ**	**1 country report**
**COLOMBIA**	**D1-D4**<br>2 desk reviews<br>70 interviews	**D1-D4**<br>Barriers and facilitators<br>**Model AAAQ**	**1 country report**

For peer review only - http://bmjopen.bmj.com/site/about/guidelines.xhtml

**HONDURAS**	**D1-D4**<br>2 desk reviews<br>70 interviews	**D1-D4**<br>Barriers and facilitators<br>**Model AAAQ**	**1 country report**
**MÉXICO**	**D1-D4**<br>2 desk reviews<br>70 interviews	**D1-D4**<br>Barriers and facilitators<br>**Model AAAQ**	**1 country report**
**URUGUAY**	**D1-D4**<br>2 desk reviews<br>70 interviews	**D1-D4**<br>Barriers and facilitators<br>**Model AAAQ**	**1 country report**
**TOTAL 5 countries**	**10 desk reviews and 350 interviews**	Barriers and facilitators per dimension in the 5 countries<br>**Model AAAQ**	**5 country reports**
**REGIONAL LEVEL**		**Synthesis exercise of the 5 country reports**	**1 regional report on lessons learnt**

<ins>Patient and public involvement</ins>

None.

Ethical considerations and dissemination

The project has been approved by the World Health Organisation Research Ethics Review Committee (ID: A66023). Moreover, it has been approved by the local Research Ethics Committee in the five participating countries: Centro Rosarino de Estudios Perinatales (Argentina), Profamilia CEIP (Colombia), Universidad Autónoma de Honduras CEIB (Honduras), Instituto Nacional de Salud Pública (México) and Centro Hospitalario Pereira Rossell (Uruguay).

The study will respect the ethical safeguards necessary to obtain informed and voluntary consent and preserve the anonymity of the key informants interviewed. Names of individuals and organizations will not be used in any final or publicly available product resulting from this research. Interviewers will be professionals with proven experience to guarantee ethical principles in conducting the interviews, date and time will be based on the participant's availability.

Data collected in this study will be managed, stored, analysed, and results will be disseminated with careful attention to protecting human subjects' rights to privacy and confidentiality. Specifically, data will be treated in compliance with and approved by the WHO's Research Ethics Review Committee (ERC) and Centro Rosarino de Estudios Perinatales Independent Ethics Committee.

For peer review only - http://bmjopen.bmj.com/site/about/guidelines.xhtml

Findings from the study will be disseminated through a multipurpose strategy to target diverse audiences so as to foster the use of the study findings to inform the public debate agenda and policy implementation at national level. The dissemination strategy will include:

-Peer-reviewed publication: Academic article(s) will be submitted to an international journal to disseminate main findings of the comparative analysis of barriers and facilitators of quality abortion policy implementation in the selected countries.

-National dissemination workshop: this workshop will gather diverse political, academic and policy actors to discuss main findings and discuss barriers and facilitators of quality abortion policy implementation.

-Regional dissemination workshop: this workshop will gather diverse political, academic and policy actors to discuss main findings of the comparative analysis of barriers and facilitators of quality abortion policy implementation in the Latin American region.

Discussion

Latin America countries have several legal and other restrictive conditions regarding abortion. However, some countries of this region also have legal norms that could allow a better access to quality abortion care but are not implemented or are ill-implemented. Interestingly, in the last years, several strategies have been adopted, which have led to improved access to abortion care, such as broad interpretation of existing regulatory frameworks and of policy governance reinforcement, as well as social pressures exerted on decision makers and politicians by women's organizations.

The WHO Global Abortion Policies Database provides a comprehensive compilation of country-specific documents regarding policies and laws. (9, 16) Existing literature has explored abortion policy implementation in various contexts, including a comparative case study that investigated health sector strategies that were useful in expanding or establishing abortion services in six countries around the world. (27) Context and actors involved in abortion reform in some Latin-American countries have also been described, (28, 29) with a recent cross-sectional study characterizing abortion-related complications, management of these complications, and women’s experiences with abortion in six countries. (30) A strength of this study is the assessment of different dimensions and actors of abortion policy implementation, which should provide a comprehensive and

For peer review only - http://bmjopen.bmj.com/site/about/guidelines.xhtml

more precise understanding of the factors that influence the implementation of these types of health policies. Nevertheless, from all complex social, legal, cultural, and ethical factors shaping abortion care and policies, this research will focus on only four to identify relevant barriers and facilitators to abortion policies, using the AAAQ framework.

Another strength of this project is that, as a multi-country study, it encompasses five countries from restrictive to more liberalised environments. This will show different contexts while looking at common patterns regarding barriers, facilitators, and strategies. This data will be collected through standardized instruments and validated by the teams involved in the project. However, there are challenges in providing a systematic comparison of heterogeneous countries. Moreover, the samples in this study may not reflect the heterogeneity of institutions and the proportionality of the population. To capture this complex scenario in each of the five country-case studies, local interview guides will be adjusted and expanded based on contextual factors.

This study will focus on factors influencing implementation and will not analyse how these factors might correspond to other aspects, such as the evaluation of abortion policies, the estimation of satisfaction with the care provided or the magnitude and severity of abortion-related complications in relation to policy implementation. Future research may consider including these and other aspects.

Finally, principal and country investigators will commit to include junior researchers in the development of the study. By conducting this research project, the coordinating team and the local teams will strengthen their skills, including those of junior researchers, to provide more responsive and sustainable policies that address women's needs and rights.

References

1. World Health Organization (WHO). Abortion care guideline. 2022. Available at: https://www.who.int/publications/i/item/9789240039483
2. World Health Organization (WHO). WHO Unsafe abortion: global and regional estimates of incidence of unsafe abortion and associated mortality in 2008. 2011. http://apps.who.int/iris/bitstream/handle/10665/44529/9789241501118_eng.pdf;jsessionid=6E4DB04A17643FA2E25D8EC75C51E609?sequence=1

For peer review only - http://bmjopen.bmj.com/site/about/guidelines.xhtml

3. Dawson A, Bateson D, Estoesta J, Sullivan E. Towards comprehensive early abortion service delivery in high income countries: insights for improving universal access to abortion in Australia. BMC Health Serv Res. 2016; 16, 612.
4. Abortion Worldwide: 20 Years of Reform. Center for Reproductive Rights. 2014. https://www.reproductiverights.org/sites/crr.civicactions.net/files/documents/20Years_Reform_Report.pdf
5. International Human Rights Law and Abortion in Latin America. Human Rights and Abortion. Human Rights Watch. 2005. https://www.hrw.org/legacy/backgrounder/wrd/wrd0106/wrd0106.pdf
6. Abortion in Latin America and the Caribbean. Fact sheet. Guttmacher Institute. 2018. https://www.guttmacher.org/sites/default/files/factsheet/fb_iaw.pdf
7. Doran F, Nancarrow S. Barriers and facilitators of access to first-trimester abortion services for women in the developed world: a systematic review. Journal of Family Planning and Reproductive Health Care. 2015; 41:170-180.
8. Abortion and Reproductive Rights in Latin America: Implications for democracy. Inter-American Dialogue and the Centre for Reproductive Rights. 2015. https://www.thedialogue.org/wp-content/uploads/2019/07/AbortionandReproductiveRightsinLatinAmerica.pdf
9. Johnson BR Jr, Lavelanet AF, Schlitt S. Global Abortion Policies Database: a new approach to strengthening knowledge on laws, policies, and human rights standards. BMC Int Health Hum Rights. 2018;12,18(1),35.
10. Dawson A, Bateson D, Estoesta J, Sullivan E. Towards comprehensive early abortion service delivery in high income countries: insights for improving universal access to abortion in Australia. BMC Health Serv Res. 2016; 16, 612.
11. Rowlands S, Lopez Arregui E. How health services can improve access to abortion. The European Journal of Contraception and Reproductive Health Care. 2016; 21, 1, 1–3

For peer review only

12. Mainey L, O'Mullan C, Reid-Searl K, Taylor A, Baird K. The role of nurses and midwives in the provision of abortion care: A scoping review. Journal of Clinical Nursing. 2020; 29(9-10), 1513–1526.
13. Olavarrieta C, Ganatra B, Sorhaindo A, Karver T, Seuc, A. et al. Nurse versus physician-provision of early medical abortion in Mexico: a randomized controlled non-inferiority trial. Bulletin of the World Health Organization. 2015; 93 (4), 249-258.
14. Ramón Michel A, Romero M, Ramos S, Gonzalez Velez C. Seguimiento de la CIPD en América Latina y el Caribe después de 2014. 2014; DOI 10.13140/RG.2.2.24107.54562.
15. World Health Organization (WHO). Gender, equity and human rights. 2016. https://www.who.int/gender-equity-rights/knowledge/AAAQ.pdf
16. Erdman JN, Johnson BR Jr. Access to knowledge and the Global Abortion Policies Database. Int J Gynaecol Obstet. 2018 ;142(1):120-124.
17. Ramón Michel A. Barreras en el acceso a los abortos legales: una mirada a las regulaciones sanitarias que incluyen el uso del misoprostol. 2013. https://clacaidigital.info/bitstream/handle/123456789/489/Misoprostol.reg.barreras.pdf?sequence=5&isAllowed=y
18. Adams WC. Conducting Semi-Structured Interviews. In: Wholey JS, Harty HP, Newcomer KE, editors. Handbook of Practical Program Evaluation. San Francisco: Jossey - Bass; 2015, p. 492–505.
19. Yin RK. Qualitative research from start to finish. The Guilford Press. 2011.
20. World Health Organization (WHO). Safe Abortion: Technical and Policy Guidance for Health Systems. 2012. https://www.who.int/reproductivehealth/publications/unsafe_abortion/9789241548434/en
21. World Health Organization (WHO). Sexual health, human rights and the law. 2015. https://apps.who.int/iris/bitstream/handle/10665/175556/9789241564984_eng.pd?sequence=1
22. Kähler L, Villumsen M, Holst Jensen M, Falk Paarup P. AAAQ & Sexual and Reproductive Health and Rights. International Indicators for Availability, Accessibility, Acceptability and Quality. The Danish

For peer review only - http://bmjopen.bmj.com/site/about/guidelines.xhtml

Institute for Human Rights. 2017. https://www.humanrights.dk/sites/humanrights.dk/files/media/migrated/aaaq-srhr_issue_paper_dihr_2017_english.pdf

23. Dennis A, Blanchard K, Bessenaar T. Identifying indicators for quality abortion care: a systematic literature review. J Fam Plann Reprod Health Care. 2017;43(1):7-15.
24. Vaismoradi, M., Turunen, H. and Bondas, T. Qualitative descriptive study. Nurs Health Sci. 2013, 15: 398-405.
25. Schmeer, K. Policy Toolkit for Strengthening Health Sector Reform. Stakeholder Analysis Guidelines. Chapter 2. Stakeholder analysis at a glance. LAC HSR. 2000.
26. Manera K, Hanson C, Gutman T, Tong A. Consensus Methods: Nominal Group Technique. In: Liamputtong P, editor. Handbook of Research Methods in Health Social Sciences. Singapore: Springer Nature; 2019, 737-750.
27. Chavkin W, Stifani BM, Bridgman-Packer D, Greenberg JM, Favier M. Implementing and expanding safe abortion care: An international comparative case study of six countries. Int J Gynecol Obstet. 2018; 143: 3-11
28. Wood S, Abracinskas L, Correa S, Pecheny M. Reform of abortion law in Uruguay: Context, process and lessons learned. Reprod Health Matters. 2016; 24:102–110
29. Ruibal A. Movement and counter-movement: A history of abortion law reform and the backlash in Colombia 2006–2014. Reprod Health Matters. 2014; 22:42–51.
30. Romero M, Gomez Ponce de Leon R, Baccaro LF, et al. Abortion related morbidity in six Latin American and Caribbean countries: findings of the WHO/HRP multi-country survey on abortion (MCS-A). BMJ Global Health 2021;6: e005618. doi:10.1136/ bmjgh-2021-005618

Authors' contributions

AL, CG, ARM, SR, GC, MR contributed to the conception and design of the study protocol, which was discussed with the rest of the authors. CG, ARM, SR, GC, MR, BC,

For peer review only

544 RGPL and AL agreed on the methodological aspects and participated in developing the
545 protocol, reviewing the draft, and making substantial revisions. CG wrote the original
546 outline. ARM together with CG were responsible of the writing, reviewing, and editing
547 the subsequent versions of the protocol. ARM oversaw developing dimension 1 section.
548 SR was in charge of developing dimension 2. MR was in charge of developing dimension
549 3. GC and CG were in charge of dimension 4. BC developed and wrote all ethics
550 considerations and prepared the original figures with SR and CG. BC and MVO revised
551 it for intellectual content and provided feedback. CG and ARM led the manuscript
552 writing. CG and ARM, with the help of MVO, coordinated the steps for article
553 development and wrote the draft of the article together. AL, SR, GC, MR and RGPL
554 contributed to manuscript writing and revision. All authors contributed to read and
555 approved the submitted version.

556 **Funding statement**

557 This project is funded by the UNDP-UNFPA-UNICEF-WHO-World Bank Special
558 Programme of Research, Development and Research Training in Human Reproduction
559 (HRP), Department of Sexual and Reproductive Health and Research, World Health
560 Organization, 20 Avenue Appia, 1211 Geneva, Switzerland. The content of this article is
561 solely the responsibility of the authors and does not reflect the views of the referred
562 programme.

563 **Competing interests**

564 The authors declare that they have no competing interests.

565 **Acknowledgements**

566 We thank the country principal investigators for their comments to the protocol and
567 contributions regarding country specific information: Edgardo Abalos (Argentina), Rocío
568 Murad (Colombia), Jakeline Álger (Honduras), Karla Berdichevsky (México), Karla
569 Flores (México) y Cecilia Stapff (Uruguay). We also thank Agostina Allori for reviewing
570 dimension 1 and Daniel Giordano for his inputs regarding data management and data
571 security aspects.

572 **Authors' details**

573 1. Centro Rosarino de Estudios Perinatales (CREP), Rosario, Argentina.

For peer review only - http://bmjopen.bmj.com/site/about/guidelines.xhtml

2. Centro de Estudios de Estado y Sociedad (CEDES), Buenos Aires, Argentina.

3. Consejo Nacional de Investigaciones Científicas y Tecnológicas (CONICET), Argentina.

4. Centro Latinoamericano de Perinatología/Salud de la Mujer y Reproductiva (CLAP/SMR) of the Pan American Health Organization (PAHO).

5. Department of Reproductive Health and Research, World Health Organization (WHO), Geneva, Switzerland.

Fig.1. Research framework: dimensions & methods

For peer review only

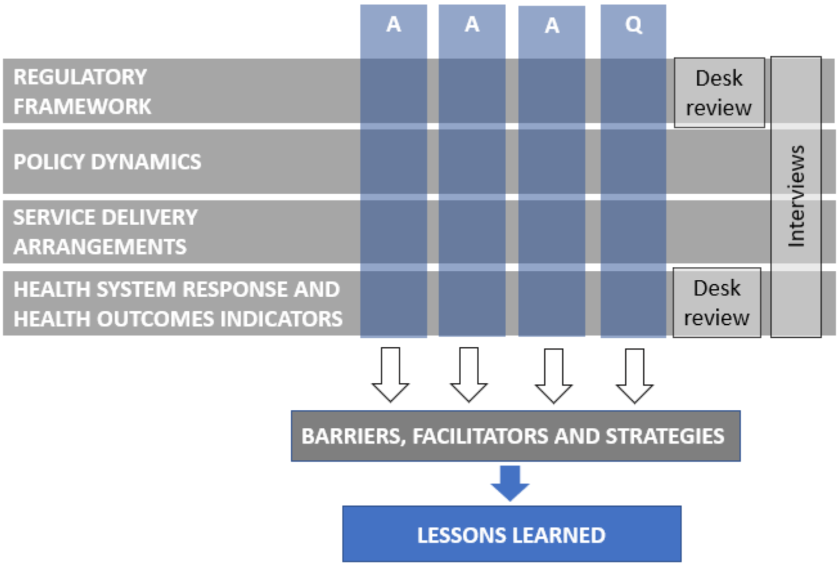

peer review only

BMJ Open

Multi-country research on comprehensive abortion policy implementation in Latin America: a mixed-methods study protocol

Journal:	*BMJ Open*
Manuscript ID	bmjopen-2023-073617.R2
Article Type:	Protocol
Date Submitted by the Author:	01-Dec-2023
Complete List of Authors:	Gialdini, Celina; Centro Rosarino de Estudios Perinatales; Blanquerna Ramon Llull University Faculty of Health Sciences<br>Ramón Michel, Agustina; CEDES<br>Romero, Mariana; CEDES; CONICET<br>Ramos, Silvina; CEDES<br>Carroli, Guillermo; CREP<br>Carroli, Berenise; CREP<br>Gomez Ponce de León, Rodolfo; Pan American Health Organization, CLAP WRH<br>Vila Ortiz, Mercedes; CREP<br>Lavelanet, Antonella ; WHO, ; World Health Organization
**Primary Subject Heading**:	Public health
Secondary Subject Heading:	Health policy, Sexual health, Obstetrics and gynaecology
Keywords:	PUBLIC HEALTH, Health policy < HEALTH SERVICES ADMINISTRATION & MANAGEMENT, SEXUAL MEDICINE, Health Services Accessibility, Organisation of health services < HEALTH SERVICES ADMINISTRATION & MANAGEMENT, Quality in health care < HEALTH SERVICES ADMINISTRATION & MANAGEMENT

SCHOLARONE™
Manuscripts

Multi-country research on comprehensive abortion policy implementation in Latin America: a mixed-methods study protocol

Authors: Gialdini, C.*[1,2], Ramón Michel, A.[3], Romero, M.[3,4], Ramos, S.[3], Carroli, G.[1], Carroli, B. [1], Gomez Ponce de León, R.[5], Vila Ortiz, M. [1], Lavelanet, A[6].

[1] Centro Rosarino de Estudios Perinatales (CREP)
[2] Blanquerna Faculty of Health Sciences, Ramon Llull University
[3] Centro de Estudios de Estado y Sociedad (CEDES)
[4] Consejo Nacional de Investigaciones Científicas y Técnicas (CONICET)
[5] Pan American Health Organization, CLAP WRH (PAHO)
[6] World Helath Organization (WHO)

*Corresponding author, cgialdini@crep.org.ar, Centro Rosarino de Estudios Perinatales

Abstract

Introduction

Access to comprehensive abortion care could prevent the death of between 13,865 and 38,940 women and the associated morbidity of five million women worldwide. There have been some important improvements in Latin America in terms of laws and policies on abortion. However, the predominant environment is still restricted and many women, adolescents, and girls still face multiple barriers to exercise their reproductive rights. This research will systematically assess comprehensive abortion policies in five Latin American countries (Argentina, Colombia, Honduras, Mexico, and Uruguay). The aim is to identify barriers, facilitators, and strategies to the implementation of abortion policies, looking at four key dimensions −regulatory framework, abortion policy dynamics, abortion service delivery, and health system and health outcomes indicators− to draw cross-cutting lessons learned to improve current implementation and inform future safe abortion policy development.

Methods and analysis

A mixed-method design will be used in the five countries to address the four dimensions through the availability, accessibility, acceptability, and quality of care model. The data collection tools include desk reviews and semi-structured interviews with key actors.

Analysis will be performed using thematic analysis and stakeholder analysis. A regional synthesis exercise will be conducted to draw lessons on barriers, facilitators, and the strategies to deal with them.

Ethics and dissemination

The project has been approved by the World Health Organisation Research Ethics Review Committee (ID: A66023) and by the local Research Ethics Committees.

Informed consent will be obtained. Data will be treated with careful attention to protecting privacy and confidentiality. Findings from the study will be disseminated through a multipurpose strategy to target diverse audiences to foster the use of the study findings to inform the public debate agenda and policy implementation at national level. The strategy will include academic, advocacy and policy arenas and actors: peer-reviewed publication, national and regional dissemination workshops.

Strengths and limitations of this study

- The study encompasses five countries with a variety of legal environments regarding abortion in order to show different contexts while looking at common patterns regarding barriers, facilitators, and strategies.
- From all complex social, legal, cultural, and ethical factors shaping abortion care and policies, this research will focus on four, using the AAAQ framework.
- This study will focus on factors influencing implementation.
- Data will be collected through standardized instruments, which might not reflect the heterogeneity among and within participating countries.

Introduction

Access to comprehensive evidence-based abortion care could prevent the death of between 13,865 and 38,940 women (1) and the associated morbidity of five million women worldwide. (2) Improving access to quality abortion care is an essential strategy for the provision of universal access to reproductive health and for the achievement of the United Nations Sustainable Development Goals (SDG): good health and well-being and gender inequality (SDGs 3 and 5). Women's access to sexual and reproductive rights can also contribute to reproductive empowerment, addressing additional SDGs such as quality education (SDG 4), sanitation and hygiene (SDG 6), decent work and economic growth (SDG 8), reducing inequalities (SDG 10) and fostering peace, justice, and strong institutions (SDG 16). (3)

For peer review only - http://bmjopen.bmj.com/site/about/guidelines.xhtml

Since the adoption of the Programme of Action (PoA) of the International Conference on Population and Development (ICPD) 28 years ago, countries have been called upon to strengthen their commitment to women's health by addressing unsafe abortion and supporting a woman's right to decide. (4) Although some improvements in terms of policies on sexual and reproductive health −including law reforms− have been achieved, many women in Latin America face multiple barriers to exercising their reproductive rights. (5)

Obstacles to quality abortion access are a matter of human rights and of public health: in this region, at least 10% of all maternal deaths are a consequence of unsafe abortions and near 760,000 women are hospitalized per year due to complications related to unsafe abortion, which violates their rights to life and health. (6) The obstacles faced by women, adolescents and girls when trying to access quality abortion include the interpretation and the implementation of legal grounds (i.e., legal indications under which is legal to have an abortion), the lack of access to information, stigma, restricted availability of health-care providers and scarcity of facilities that can lawfully provide services, among others. (7) These barriers can disproportionately affect specific groups, such as adolescents, women that are poor, indigenous women, women living in small towns, and other women in vulnerable social conditions.

In this context, reforming abortion laws has proven to be challenging. Most Latin American countries have restrictive abortion laws, allowing legal termination of pregnancy only under certain grounds such as rape, severe fetal impairment or when the woman's life or health is at risk. (8) Abortion is legal without restriction as to reason in the first trimester of pregnancy in Argentina, Cuba, Uruguay, and nine states of Mexico (Mexico City, Oaxaca, Hidalgo, Veracruz, Baja California, Baja California Sur, Sinaola, Guerrero and Colima). Moreover, in February 2022, the Colombian Constitutional Court ruled to decriminalize abortion up to 24 weeks of pregnancy.

Despite a restricted environment, some countries have managed to enhance the availability, accessibility, acceptability and quality of abortion services and care by resorting to alternative strategies. Court judgments and public health guidelines have been used to expand the interpretation of the circumstances under which abortion is

For peer review only

allowed. In settings with very restrictive legal environments, like Honduras, guidelines to manage post-abortion care have also been issued. (9)

Taking this regional context into consideration, we will systematically look at comprehensive abortion policies in five Latin American countries −Argentina, Colombia, Honduras, Mexico, and Uruguay− to understand how context, content, actors and processes affect the implementation of comprehensive abortion policies. The aim is to assess abortion regulations[1] and their use in practice, the policy dynamics, the service delivery arrangements, and the abortion-related health indicators and how they are monitored and evaluated, to identify barriers and facilitators to the implementation of comprehensive abortion policy and the strategies used to address them in each country. Finally, we will identify similarities and differences among barriers, facilitators, and strategies of policy implementation across countries, and draw lessons learned from these implementation processes and the strategies to improve access to evidence-based abortion care.

Methods and analysis

Design and framework

In this research study we will look at the context, content, actors, and processes affecting the implementation and monitoring of comprehensive abortion policies and comprises five country-case studies and a regional synthesis of findings. We will use a mixed-method design applying diverse data gathering techniques and analysis strategies to assess four key dimensions of abortion policy[2]:

- The **regulatory framework** (dimension 1)
- The **policy dynamics** (dimension 2)
- The **service delivery arrangements** (dimension 3)
- The **health system and health outcomes indicators** (dimension 4)

[1] By Regulations we mean rules or guidelines established and enforced by an authority such as constitutional clauses, laws, decrees, and other sort of administrative regulations

[2] By Policy we mean a system of laws, regulatory measures, courses of action, and funding priorities concerning a given topic promulgated, in this case, abortion.

For peer review only - http://bmjopen.bmj.com/site/about/guidelines.xhtml

In dimension 1 (regulatory framework) we will assess how and to what extent regulations −both written and in practice− pose as barriers and facilitators to quality abortion access, and the strategies to deal with them. We will address the traits and use of existing abortion related regulations. This dimension encompasses laws, resolutions, decrees, health standards and health guidelines, technical guidelines, and other official documents with or without legal status. First, we will perform a descriptive analysis of the regulatory framework, and then an examination of the practice.

In dimension 2 (policy dynamics) we will assess how aspects such as agenda setting, design and implementation processes, and governance and actors' involvement, pose as barriers and facilitators of abortion policy, and how they are addressed through different strategies. We will assess the agenda setting looking at the political and social conditions that place abortion policy on the public agenda. For design and implementation aspects we will focus on the shaping of content in policy formulation and on the main challenges of implementation. Governance issues include the capacity of health authorities to rule, reinforce and have control of the implementation of abortion policy for the provision and access to comprehensive abortion care. We will address the policy dynamics from the perspective of policy makers, health services managers and women's organizations.

In dimension 3 (service delivery arrangements) we will assess how health services are organised to ensure access to quality abortion care, and how barriers and facilitators are addressed by different strategies. (10-14)

In dimension 4 (health system and health outcomes indicators) we will assess what information on health system and health outcomes indicators related to abortion care are monitored and evaluated. We will address the process of generating, collecting, reporting, monitoring, and evaluating health system and health outcomes indicators, and how this evaluation accounts for policy implementation progress and quality of care oversight.

We will assess these dimensions through availability, accessibility, acceptability, and quality of care as framed in the **AAAQ model**. We will use this framework to gather and systematize the data coming from the two data collection instruments: semi-structured interviews and document review. *Availability* refers to the need of having enough functioning public health and health care facilities, goods, and services. *Accessibility* refers to the need of making all those facilities, goods, and services physically accessible and affordable to everyone within the jurisdiction of the State party without

For peer review only

discrimination. *Acceptability* refers to the need of the health system responses to be respectful of the law, medical ethics, and to be culturally appropriate and sensitive to gender and age. *Quality of care* also refers to the need for health goods and services to be scientifically and medically approved and of good quality following WHO standards. (15) Specifically, *quality of abortion care* refers to the fact that the provision of abortion services should be effective, efficient, accessible, acceptable (patient centred), equitable and safe. (1)

As a result of the appraisal of the four dimensions, we will identify the **barriers and facilitators** to availability, accessibility, acceptability, and quality of care regarding abortion related to abortion policy implementation.

Finally, we will synthesize findings from the case studies to draw lessons learned on cross-cutting barriers and facilitators, as well as the similarities and differences on how comprehensive abortion policies are implemented in the participant countries. These lessons learned can be used to inform future decision-making across the region and other sociocultural and geopolitical contexts. (See Fig. 1).

For this study, comprehensive abortion care includes the elements of induced abortion under legal indications as well as all the elements of post abortion care: information, counselling, treatment, contraceptive services, reproductive health services, community, and service providers' partnerships.

Settings

We will conduct case-studies in five selected countries: Argentina, Colombia, Honduras, Mexico, and Uruguay. The selection of settings addresses diversity in abortion regulations as well as diversity in countries' institutional political organization across a continuum of abortion related legal access. We aim to include countries going from an absolute legal restrictive environment with only post-abortion care provision to countries with less restrictive legal status of abortion, availability of abortion guidelines, or registration and mifepristone and misoprostol availability, two medicines used in the medical abortion process. The selection also considers common issues on abortion policy

For peer review only - http://bmjopen.bmj.com/site/about/guidelines.xhtml

implementation and indicators among countries. Country profiles are summarized in Table 1.

Table 1: Country profile

DIMENSIONS \ COUNTRIES	Argentina	Colombia	Honduras	México[3]	Uruguay
**Socio economic profile**	- 42% of the population lives under poverty[4]<br>- 42, 3 Gini Coefficient[5]<br>- High level of Inflation. The second country with highest (triple digit inflation)<br>- A historically large middle class is shrinking	- 42% of the population lives under poverty[6]<br>-54, 2 Gini Coefficient[7]<br>- Under a peace agreement process, with still sites with high levels of violence and isolation	- 48% of the population lives under poverty[8]<br>- 48, 2 Gini Coefficient[9]<br>- The size of the middle class was among the smallest in the region (18% compared to an average 41%)<br>- High levels of violence: over 38 homicides per 100,000 inhabitants	- 44% of the population lives under poverty[10]<br>- 45, 4 Gini Coefficient[11]<br>- High levels of social and political violence connected with the illegal drug market	- 11% of the population lives under poverty[12]<br>- 40, 3 Gini Coefficient[13]<br>- Its middle class is the largest in the Americas, representing more than 60% of its population[14]
**Division of the geographical power**	Federalism with shared competences between national and local government	Centralist with local powers	Unitarian	Strong Federalism	Centralist
**Abortion legal model**	Mixed (indications and voluntary interruption of pregnancy) with partial decriminalization	Mixed with total decriminalized up to 24 weeks	Total ban	Mixed at some states<br><br>Indication model in other states.	Mixed with partial decriminalization

For peer review only

---

[3] Mexico has different regulation it each state, so the references here are regarding the national level

[4] World Bank, 'Pobreza en América Latina', *Statista*, 2022, https://es.statista.com/temas/8701/pobreza-en-america-latina/.

[5] World Bank, 'Países con mayor desigualdad en el coeficiente Gini en América Latina', *Statista*, 2021, https://es.statista.com/estadisticas/1267584/latinoamerica-coeficiente-gini-desigualdad-de-ingresos-por-pais/..

[6] World Bank, 'Pobreza en América Latina', op. cit. 2.

[7] World Bank, 'Países con mayor desigualdad en el coeficiente Gini en América Latina', op. cit. 3.

[8] World Bank, 'Pobreza en América Latina', op. cit. 2.

[9] World Bank, 'Países con mayor desigualdad en el coeficiente Gini en América Latina', op. cit. 3.

[10] World Bank, 'Pobreza en América Latina', op. cit. 2.

[11] World Bank, 'Países con mayor desigualdad en el coeficiente Gini en América Latina', op. cit. 3.

[12] World Bank, 'Pobreza en América Latina', op. cit. 2.

[13] World Bank, 'Países con mayor desigualdad en el coeficiente Gini en América Latina', op. cit. 3.

[14] World Bank, 'Uruguay: panorama general', *World Bank*, 2023, https://www.bancomundial.org/es/country/uruguay/overview.

**Main characteristics of abortion regulation**	-The law allows any health professional to provide abortion services<br>-No rape report is required for access legal abortion under the rape indication<br>-There is no abortion committee<br>-Conscientious objection is allowed but with limitations and only at an individual level	-The law allows any health professional to provide abortion services<br>-There is no abortion committee<br>-Ambulatory abortion is accepted<br>-No rape report is required for access legal abortion under the rape indication<br>-Conscientious objection is allowed but with limitations and only at an individual level	----------	-In some states rape report is required<br>-There is no abortion committee<br>-Conscientious objection is allowed but with limitations and only at an individual level at the national level but there is still	-There is an abortion committee<br>-Medical based regulation, with several other requirements<br>-Conscientious objection is allowed in broad terms, both individual and institutional
**Protocol of safe and legal abortion**	Yes<br>National	Yes<br>National	No<br>Just Protocol for Management of Obstetric Complication	Yes<br>Locals with variations	Yes<br>National
**Institutional adjudication**	Congress	Court	----------	Local legislatures	Congress
**Level where care is provided**	First and second level	First and second level	Second level	First and second level	Second level
**Health service arrangements**	Public health sector is the main abortion provider together with the community sector (also called acompañantes or informal health system)	Private clinics (Pro Familia and Orientame) are two of the main abortion providers	Some Public hospital are the ones proving abortion care	Public specialized abortion clinics are the main providers in México City	Public health sector is the main provider, and private clinics are not promoted

<u>Data collection and tools</u>

We will utilize two data collection methods: desk review (for dimensions 1 and 4) and semi-structured interviews (for dimensions 1, 2, 3, and 4). The data collection process is summarized in Table 2. We will use the quantitative data not only to inform the qualitative process, but to integrate it into the analysis to better elucidate different aspects of the dimensions, thereby allowing for a robust process of triangulation.

Desk review

For dimension 1, we will review the texts of regulations to identify and categorize abortion related regulations, we will structure a data extraction matrix into two domains:

− the abortion regulatory framework, which refers to the specific regulations and practices related to abortion.

− the legal environment, which includes regulations that are not specifically about abortion but have a direct impact on access to abortion health services (e.g., laws regarding patient confidentiality, norms regarding minors' consent, norms on mental or intellectual disabilities, laws related to sexual violence).

For dimension 4, we will conduct a systematic search for country-specific statistics, reports and documents. National official reports and databases containing processes,

For peer review only - http://bmjopen.bmj.com/site/about/guidelines.xhtml

outputs, or outcome indicators relevant to abortion care at national level will be reviewed and gaps in indicator reporting, monitoring and evaluation will be identified.

We will base the data extraction matrix for dimension 1, on the topics included in the WHO Global Abortion Policies Database (9, 16) and in the report "*Barreras en el acceso a los abortos legales: una mirada a las regulaciones sanitarias que incluyen el uso del misoprostol*". (17) For dimension 4, we will use a data extraction matrix structured with the AAAQ domains to identify and categorize abortion related indicators. From each reported indicator, we will collect data on indicator definition, method of measurement, data collection methodology and frequency, date of publication, source of information and indicator value. If data are stratified (e.g., race, gender, age, urban/rural), this information will also be collected.

Semi-structured interviews

We will conduct semi-structured interviews (18) to explore:

- how legal and regulatory instruments are used, how they pose as barriers and facilitators to abortion service provision and access, and how regulations are translated into practice (dimension 1).
- the policy dynamics related to agenda setting, governance issues, financing schemes, and actors' involvement (dimension 2).
- which are the service arrangements adopted according to the different settings, human resources, and the technologies available, and which were the triggers for the model/different models currently used (dimension 3); and
- which approaches each country uses for monitoring abortion-related care and for the oversight of maternal mortality, near miss and quality of care and how monitoring and evaluation of process –and outcomes– indicators related to abortion are operationalized (dimension 4).

An experienced interviewer with social science or public health background with legal or programmatic knowledge will conduct the interviews. The interviewers will obtain informed consent, conduct the interviews in person or through digital technology, and record and transcribe the interviews.

Participants

For peer review only - http://bmjopen.bmj.com/site/about/guidelines.xhtml

For the interviews, we will use a purposive sample of twenty key informants for dimensions 1 to 3 and 10 key informants for dimension 4 in each country. We seek to obtain a broad range of perspectives on the subject (19). The selected subjects will be key informants in the abortion policy domain, encompassing different categories: health providers, sexual and reproductive health policy decision makers, researchers, leaders of women's organizations, judicial agents, politicians, opinion leaders, etc.

The sample size is a target overall number, which we will adjust during the data collection process. We consider that twenty key informants for dimension 1 to 3 and ten key informants for dimension 4 in each country is feasible sample size that should allow for variability along the different categories (see Table 1).

The identification of participants is expected to be different depending on how broad or narrow the categories or levels from where they are identified are. The criteria of variability of forums or levels aims at obtaining diversity and having those that yield the most plentiful data in the matter.

We will identify the key informants through a mapping of actors. A first round of mapping will be based on teams' expertise and networks. We expect to easily identify narrow-level participants such as decision makers (who are few in each national abortion domain). For broad categories, such as health providers and activists (who are many and disperse in different settings), we will ultimately define the selection strategy with each country's principal investigator to achieve a convenience sample that accounts for variability. There may be a second round of mapping to select knowledgeable stakeholders identified by the interviewees from the first round.

Table 2 Data collection

	**Dimension 1**	**Dimension 2**	**Dimension 3**	**Dimension 4**	**Total**
**ARGENTINA**	Desk review<br>20 interviews	20 interviews	20 interviews	Desk review<br>10 interviews	2 desk reviews<br>70 interviews
**COLOMBIA**	**Dimension 1**	**Dimension 2**	**Dimension 3**	**Dimension 4**	**Total**
	Desk review<br>20 interviews	20 interviews	20 interviews	Desk review<br>10 interviews	2 desk reviews<br>70 interviews
**HONDURAS**	**Dimension 1**	**Dimension 2**	**Dimension 3**	**Dimension 4**	**Total**

For peer review only

For peer review only - http://bmjopen.bmj.com/site/about/guidelines.xhtml

	Desk review<br>20 interviews	20 interviews	20 interviews	Desk review<br>10 interviews	2 desk reviews<br>70 interviews
**MÉXICO**	**Dimension 1**	**Dimension 2**	**Dimension 3**	**Dimension 4**	**Total**
	Desk review<br>20 interviews	20 interviews	20 interviews	Desk review<br>10 interviews	2 desk reviews<br>70 interviews
**URUGUAY**	**Dimension 1**	**Dimension 2**	**Dimension 3**	**Dimension 4**	**Total**
	Desk review<br>20 interviews	20 interviews	20 interviews	Desk review<br>10 interviews	2 desk reviews<br>70 interviews
**TOTAL 5 countries**	**Dimension 1**	**Dimension 2**	**Dimension 3**	**Dimension 4**	**TOTAL**
	5 desk reviews<br>100 interviews	100 interviews	100 interviews	5 desk reviews<br>50 interviews	**10 desk reviews**<br>**350 interviews**

Analysis

Document review data analysis (each country study)

For dimension 1, we will chart and narratively synthesize data from the document review based on the explicit wording and content of the text, following the categories of the WHO Global Abortion Policies Database. (9, 16). This WHO database contains information related to authorization and service-delivery requirements, conscientious objection, penalties, national SRH indicators, and UN Treaty Monitoring Body concluding observations among other documents.

The written words of the documents, however, take their meaning from their purpose and context, and often require interpretation. Therefore, for the interpretation of competing or inconsistent legal primary sources, legal secondary sources will be used. Additionally, we will carry out a preliminary classification as barrier or facilitator for each topic for the purpose to assess them in the interviews.

For dimension 4, we will synthesize data from the document review and further collect indicators for abortion care; we will classify and analyse this data according to the AAAQ domains. The data extraction matrix will also be based on indicators for abortion care as identified as part of the WHO 2022 indicators for abortion, the Danish Institute for Human Rights reports on sexual and reproductive health and rights and the systematic review by Dennis *et.al.* (20-23) We will identify gaps between the extraction matrix and the available indicators.

For peer review only

We will use data from the desk reviews to gain a comprehensive understanding of the subject matter and identify key themes or gaps in knowledge. We will also use country specific statistics and documents to inform the interviews.

Interview data analysis (country studies)

We will use an inductive thematic coding approach with the data from the interviews for each dimension and organize a matrix round central aspects of the corresponding dimension. A content analysis will follow the coding to identify emerged themes and patterns among them. (24) We will identify barriers and facilitators of policy implementation in relation to the AAAQ framework in a data reduction matrix. The first step will be to extract the data from the interviews and place it within the topics of the data extraction matrix. Then, a second reading of the data in the matrix will take place, guided by specific questions for each dimension. Finally, the analysis of barriers, facilitators and strategies will be organized under the AAAQ matrix.

We will conduct a stakeholder analysis to map the actors who have had an interest and have affected or influenced the implementation of abortion policy (dimension 2), to better understand their role in the continuum supporters-blockers, following the questions suggested by Schmeer (25):

- Who are the most important abortion stakeholders (from a power and leadership perspective)?
- What are the different stakeholders' positions on abortion policies?
- What do the different stakeholders see as possible advantages or disadvantages of abortion policies?
- Which stakeholders form alliances? Is there potential for new alliances to form?

Identification and synthesis of barriers, facilitators, and strategies of comprehensive abortion policy implementation *(country studies)*

We will then apply the findings from dimensions 1 to 4 to a data grid organised with the AAAQ framework to better illustrate the barriers, facilitators, and strategies to deal with them. This aims to group the information from the four dimensions in one single document, which will shed light on strategies used towards comprehensive abortion policy implementation. (Fig.1)

For peer review only - http://bmjopen.bmj.com/site/about/guidelines.xhtml

We will analyse this information using substantive themes (or codes or clusters of codes). The rearrangements and recombination may be facilitated by depicting the data graphically or by arranging them in lists, for example.

The collection of quantitative data will provide a quantifiable framework to evaluate barriers and facilitators in abortion care access and quality. We will obtain a holistic view by integrating quantitative and qualitative data, addressing both the qualitative depth of policy experiences and the quantitative breadth of their impact on abortion care policy.

Lessons learned from the five country studies (regional synthesis)

At this final stage of the study, we will conduct a regional synthesis exercise to draw lessons on barriers, facilitators and the strategies provided by the five country studies. Once the first version of this synthesis exercise is ready, we will conduct a validation process involving members of all five national teams to reach consensus on the synthesis. For this validation, we will apply an adjusted nominal group technique. (26)

Summary of study process

We expect to start the study in February 2024 and finish it in May 2025. The study process is summarized in Table 3:

Table 3 Study process

COUNTRIES	DATA COLLECTION	DATA ANALYSIS	FINAL PRODUCT
**ARGENTINA**	**D1-D4**<br>2 desk reviews<br>70 interviews	**D1-D4**<br>Barriers and facilitators<br>**Model AAAQ**	**1 country report**
**COLOMBIA**	**D1-D4**<br>2 desk reviews<br>70 interviews	**D1-D4**<br>Barriers and facilitators<br>**Model AAAQ**	**1 country report**
**HONDURAS**	**D1-D4**<br>2 desk reviews<br>70 interviews	**D1-D4**<br>Barriers and facilitators<br>**Model AAAQ**	**1 country report**
**MÉXICO**	**D1-D4**<br>2 desk reviews<br>70 interviews	**D1-D4**<br>Barriers and facilitators<br>**Model AAAQ**	**1 country report**
**URUGUAY**	**D1-D4**<br>2 desk reviews<br>70 interviews	**D1-D4**<br>Barriers and facilitators<br>**Model AAAQ**	**1 country report**
**TOTAL 5 countries**	**10 desk reviews and 350 interviews**	Barriers and facilitators per dimension in the 5 countries<br>**Model AAAQ**	**5 country reports**

For peer review only

REGIONAL LEVEL		Synthesis exercise of the 5 country reports	1 regional report on lessons learnt

Positionality statement

We are researchers working in the field of abortion policy implementation, sexual and reproductive rights and evidence-based practices living in Argentina. We will work on making sure that any biases or assumptions we have or we may bring are documented.

Patient and public involvement

None.

Ethics and dissemination

The project has been approved by the World Health Organisation Research Ethics Review Committee (ID: A66023). Moreover, it has been approved by the local Research Ethics Committee in the five participating countries: Centro Rosarino de Estudios Perinatales (Argentina), Profamilia CEIP (Colombia), Universidad Autónoma de Honduras CEIB (Honduras), Instituto Nacional de Salud Pública (México) and Centro Hospitalario Pereira Rossell (Uruguay).

The study will respect the ethical safeguards necessary to obtain informed and voluntary consent and preserve the anonymity of the key informants interviewed. Names of individuals and organizations will not be used in any final or publicly available product resulting from this research. Interviewers will be professionals with proven experience to guarantee ethical principles in conducting the interviews, date and time will be based on the participant's availability.

Data collected in this study will be managed, stored, analysed, and results will be disseminated with careful attention to protecting human subjects' rights to privacy and confidentiality. Specifically, data will be treated in compliance with and approved by the WHO's Research Ethics Review Committee (ERC) and Centro Rosarino de Estudios Perinatales Independent Ethics Committee.

Findings from the study will be disseminated through a multipurpose strategy to target diverse audiences so as to foster the use of the study findings to inform the public debate agenda and policy implementation at national level. The dissemination strategy will include:

For peer review only - http://bmjopen.bmj.com/site/about/guidelines.xhtml

-Peer-reviewed publication: Academic article(s) will be submitted to an international journal to disseminate main findings of the comparative analysis of barriers and facilitators of quality abortion policy implementation in the selected countries.

-National dissemination workshop: this workshop will gather diverse political, academic and policy actors to discuss main findings and discuss barriers and facilitators of quality abortion policy implementation.

-Regional dissemination workshop: this workshop will gather diverse political, academic and policy actors to discuss main findings of the comparative analysis of barriers and facilitators of quality abortion policy implementation in the Latin American region.

Discussion

Latin America countries have several legal and other restrictive conditions regarding abortion. However, some countries of this region also have legal norms that could allow a better access to quality abortion care but are not implemented or are ill-implemented. Interestingly, in the last years, several strategies have been adopted, which have led to improved access to abortion care, such as broad interpretation of existing regulatory frameworks and of policy governance reinforcement, as well as social pressures exerted on decision makers and politicians by women's organizations.

The WHO Global Abortion Policies Database provides a comprehensive compilation of country-specific documents regarding policies and laws. (9, 16) Existing literature has explored abortion policy implementation in various contexts, including a comparative case study that investigated health sector strategies that were useful in expanding or establishing abortion services in six countries around the world. (27) Context and actors involved in abortion reform in some Latin-American countries have also been described, (28, 29) with a recent cross-sectional study characterizing abortion-related complications, management of these complications, and women’s experiences with abortion in six countries. (30) A strength of this study is the assessment of different dimensions and actors of abortion policy implementation, which should provide a comprehensive and more precise understanding of the factors that influence the implementation of these types of health policies. Nevertheless, from all complex social, legal, cultural, and ethical factors shaping abortion care and policies, this research will focus on only four to identify relevant barriers and facilitators to abortion policies, using the AAAQ framework.

For peer review only

Another strength of this project is that, as a multi-country study, it encompasses five countries from restrictive to more liberalised environments. This will show different contexts while looking at common patterns regarding barriers, facilitators, and strategies. This data will be collected through standardized instruments and validated by the teams involved in the project. However, there are challenges in providing a systematic comparison of heterogeneous countries. Moreover, the samples in this study may not reflect the heterogeneity of institutions and the proportionality of the population. To capture this complex scenario in each of the five country-case studies, local interview guides will be adjusted and expanded based on contextual factors.

This study will focus on factors influencing implementation and will not analyse how these factors might correspond to other aspects, such as the evaluation of abortion policies, the estimation of satisfaction with the care provided or the magnitude and severity of abortion-related complications in relation to policy implementation. Future research may consider including these and other aspects.

Finally, principal and country investigators will commit to include junior researchers in the development of the study. By conducting this research project, the coordinating team and the local teams will strengthen their skills, including those of junior researchers, to provide more responsive and sustainable policies that address women's needs and rights.

Contributors

AL, CG, ARM, SR, GC, MR contributed to the conception and design of the study protocol, which was discussed with the rest of the authors. CG, ARM, SR, GC, MR, BC, RGPL and AL agreed on the methodological aspects and participated in developing the protocol, reviewing the draft, and making substantial revisions. CG wrote the original outline. ARM together with CG were responsible of the writing, reviewing, and editing the subsequent versions of the protocol. ARM oversaw developing dimension 1 section. SR was in charge of developing dimension 2. MR was in charge of developing dimension 3. GC and CG were in charge of dimension 4. BC developed and wrote all ethics considerations and prepared the original figures with SR and CG. BC and MVO revised it for intellectual content and provided feedback. CG and ARM led the manuscript writing. CG and ARM, with the help of MVO, coordinated the steps for article development and wrote the draft of the article together. AL, SR, GC, MR and RGPL

For peer review only - http://bmjopen.bmj.com/site/about/guidelines.xhtml

contributed to manuscript writing and revision. All authors contributed to read and approved the submitted version.

Funding

This project is funded by the UNDP-UNFPA-UNICEF-WHO-World Bank Special Programme of Research, Development and Research Training in Human Reproduction (HRP), Department of Sexual and Reproductive Health and Research, World Health Organization, 20 Avenue Appia, 1211 Geneva, Switzerland. The content of this article is solely the responsibility of the authors and does not reflect the views of the referred programme.

Competing interests

The authors declare that they have no competing interests.

Acknowledgements

We thank the country principal investigators for their comments to the protocol and contributions regarding country specific information: Edgardo Abalos (Argentina), Rocío Murad (Colombia), Jakeline Álger (Honduras), Karla Berdichevsky (México), Karla Flores (México) y Cecilia Stapff (Uruguay). We also thank Agostina Allori for reviewing dimension 1 and Daniel Giordano for his inputs regarding data management and data security aspects.

Authors' details

1. Centro Rosarino de Estudios Perinatales (CREP), Rosario, Argentina.

2. Centro de Estudios de Estado y Sociedad (CEDES), Buenos Aires, Argentina.

3. Consejo Nacional de Investigaciones Científicas y Tecnológicas (CONICET), Argentina.

4. Centro Latinoamericano de Perinatología/Salud de la Mujer y Reproductiva (CLAP/SMR) of the Pan American Health Organization (PAHO).

5. Department of Reproductive Health and Research, World Health Organization (WHO), Geneva, Switzerland.

For peer review only

References

1. World Health Organization (WHO). Abortion care guideline. 2022. Available at: https://www.who.int/publications/i/item/9789240039483
2. World Health Organization (WHO). WHO Unsafe abortion: global and regional estimates of incidence of unsafe abortion and associated mortality in 2008. 2011. http://apps.who.int/iris/bitstream/handle/10665/44529/9789241501118_eng.pdf;jsessionid=6E4DB04A17643FA2E25D8EC75C51E609?sequence=1
3. Dawson A, Bateson D, Estoesta J, Sullivan E. Towards comprehensive early abortion service delivery in high income countries: insights for improving universal access to abortion in Australia. BMC Health Serv Res. 2016; 16, 612.
4. Abortion Worldwide: 20 Years of Reform. Center for Reproductive Rights. 2014. https://www.reproductiverights.org/sites/crr.civicactions.net/files/documents/20Years_Reform_Report.pdf
5. International Human Rights Law and Abortion in Latin America. Human Rights and Abortion. Human Rights Watch. 2005. https://www.hrw.org/legacy/backgrounder/wrd/wrd0106/wrd0106.pdf
6. Abortion in Latin America and the Caribbean. Fact sheet. Guttmacher Institute. 2018. https://www.guttmacher.org/sites/default/files/factsheet/fb_iaw.pdf
7. Doran F, Nancarrow S. Barriers and facilitators of access to first-trimester abortion services for women in the developed world: a systematic review. Journal of Family Planning and Reproductive Health Care. 2015; 41:170-180.
8. Abortion and Reproductive Rights in Latin America: Implications for democracy. Inter-American Dialogue and the Centre for Reproductive Rights. 2015. https://www.thedialogue.org/wp-content/uploads/2019/07/AbortionandReproductiveRightsinLatinAmerica.pdf

For peer review only - http://bmjopen.bmj.com/site/about/guidelines.xhtml

9. Johnson BR Jr, Lavelanet AF, Schlitt S. Global Abortion Policies Database: a new approach to strengthening knowledge on laws, policies, and human rights standards. BMC Int Health Hum Rights. 2018;12,18(1),35.

10. Dawson A, Bateson D, Estoesta J, Sullivan E. Towards comprehensive early abortion service delivery in high income countries: insights for improving universal access to abortion in Australia. BMC Health Serv Res. 2016; 16, 612.

11. Rowlands S, Lopez Arregui E. How health services can improve access to abortion. The European Journal of Contraception and Reproductive Health Care. 2016; 21, 1, 1–3

12. Mainey L, O'Mullan C, Reid-Searl K, Taylor A, Baird K. The role of nurses and midwives in the provision of abortion care: A scoping review. Journal of Clinical Nursing. 2020; 29(9-10), 1513–1526.

13. Olavarrieta C, Ganatra B, Sorhaindo A, Karver T, Seuc, A. et al. Nurse versus physician-provision of early medical abortion in Mexico: a randomized controlled non-inferiority trial. Bulletin of the World Health Organization. 2015; 93 (4), 249-258.

14. Ramón Michel A, Romero M, Ramos S, Gonzalez Velez C. Seguimiento de la CIPD en América Latina y el Caribe después de 2014. 2014; DOI 10.13140/RG.2.2.24107.54562.

15. World Health Organization (WHO). Gender, equity and human rights. 2016. https://www.who.int/gender-equity-rights/knowledge/AAAQ.pdf

16. Erdman JN, Johnson BR Jr. Access to knowledge and the Global Abortion Policies Database. Int J Gynaecol Obstet. 2018 ;142(1):120-124.

17. Ramón Michel A. Barreras en el acceso a los abortos legales: una mirada a las regulaciones sanitarias que incluyen el uso del misoprostol. 2013. https://clacaidigital.info/bitstream/handle/123456789/489/Misoprostol.reg.barreras.pdf?sequence=5&isAllowed=y

18. Adams WC. Conducting Semi-Structured Interviews. In: Wholey JS, Harty HP, Newcomer KE, editors. Handbook of Practical Program Evaluation. San Francisco: Jossey - Bass; 2015, p. 492–505.

19. Yin RK. Qualitative research from start to finish. The Guilford Press. 2011.

For peer review only

20. World Health Organization (WHO). Safe Abortion: Technical and Policy Guidance for Health Systems. 2012. https://www.who.int/reproductivehealth/publications/unsafe_abortion/9789241548434/en
21. World Health Organization (WHO). Sexual health, human rights and the law. 2015. https://apps.who.int/iris/bitstream/handle/10665/175556/9789241564984_eng.pd?sequence=1
22. Kähler L, Villumsen M, Holst Jensen M, Falk Paarup P. AAAQ & Sexual and Reproductive Health and Rights. International Indicators for Availability, Accessibility, Acceptability and Quality. The Danish Institute for Human Rights. 2017. https://www.humanrights.dk/sites/humanrights.dk/files/media/migrated/aaaq-srhr_issue_paper_dihr_2017_english.pdf
23. Dennis A, Blanchard K, Bessenaar T. Identifying indicators for quality abortion care: a systematic literature review. J Fam Plann Reprod Health Care. 2017;43(1):7-15.
24. Vaismoradi, M., Turunen, H. and Bondas, T. Qualitative descriptive study. Nurs Health Sci. 2013, 15: 398-405.
25. Schmeer, K. Policy Toolkit for Strengthening Health Sector Reform. Stakeholder Analysis Guidelines. Chapter 2. Stakeholder analysis at a glance. LAC HSR. 2000.
26. Manera K, Hanson C, Gutman T, Tong A. Consensus Methods: Nominal Group Technique. In: Liamputtong P, editor. Handbook of Research Methods in Health Social Sciences. Singapore: Springer Nature; 2019, 737-750.
27. Chavkin W, Stifani BM, Bridgman-Packer D, Greenberg JM, Favier M. Implementing and expanding safe abortion care: An international comparative case study of six countries. Int J Gynecol Obstet. 2018; 143: 3-11
28. Wood S, Abracinskas L, Correa S, Pecheny M. Reform of abortion law in Uruguay: Context, process and lessons learned. Reprod Health Matters. 2016; 24:102–110

For peer review only - http://bmjopen.bmj.com/site/about/guidelines.xhtml

29. Ruibal A. Movement and counter-movement: A history of abortion law reform and the backlash in Colombia 2006–2014. Reprod Health Matters. 2014; 22:42–51.
30. Romero M, Gomez Ponce de Leon R, Baccaro LF, et al. Abortion related morbidity in six Latin American and Caribbean countries: findings of the WHO/HRP multi-country survey on abortion (MCS-A). BMJ Global Health 2021;6: e005618. doi:10.1136/ bmjgh-2021-005618

Fig.1. Research framework: dimensions & methods

For peer review only

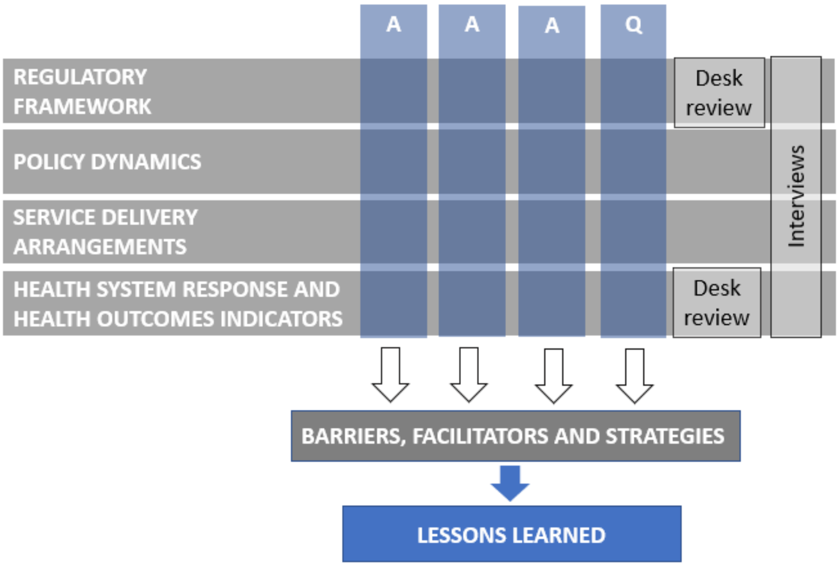

peer review only

BMJ Open

Multi-country research on comprehensive abortion policy implementation in Latin America: a mixed-methods study protocol

Journal:	*BMJ Open*
Manuscript ID	bmjopen-2023-073617.R3
Article Type:	Protocol
Date Submitted by the Author:	06-Dec-2023
Complete List of Authors:	Gialdini, Celina; Centro Rosarino de Estudios Perinatales; Blanquerna Ramon Llull University Faculty of Health Sciences<br>Ramón Michel, Agustina; CEDES<br>Romero, Mariana; CEDES; CONICET<br>Ramos, Silvina; CEDES<br>Carroli, Guillermo; CREP<br>Carroli, Berenise; CREP<br>Gomez Ponce de León, Rodolfo; Pan American Health Organization, CLAP WRH<br>Vila Ortiz, Mercedes; CREP<br>Lavelanet, Antonella ; WHO, ; World Health Organization
**Primary Subject Heading**:	Public health
Secondary Subject Heading:	Health policy, Sexual health, Obstetrics and gynaecology
Keywords:	PUBLIC HEALTH, Health policy < HEALTH SERVICES ADMINISTRATION & MANAGEMENT, SEXUAL MEDICINE, Health Services Accessibility, Organisation of health services < HEALTH SERVICES ADMINISTRATION & MANAGEMENT, Quality in health care < HEALTH SERVICES ADMINISTRATION & MANAGEMENT

SCHOLARONE™
Manuscripts

Multi-country research on comprehensive abortion policy implementation in Latin America: a mixed-methods study protocol

Gialdini, C.*[1,2], Ramón Michel, A.[3], Romero, M.[3,4], Ramos, S.[3], Carroli, G.[1], Carroli, B. [1], Gomez Ponce de León, R.[5], Vila Ortiz, M. [1], Lavelanet, A[6].

[1] Centro Rosarino de Estudios Perinatales (CREP)
[2] Blanquerna Faculty of Health Sciences, Ramon Llull University
[3] Centro de Estudios de Estado y Sociedad (CEDES)
[4] Consejo Nacional de Investigaciones Científicas y Técnicas (CONICET)
[5] Pan American Health Organization, CLAP WRH (PAHO)
[6] World Helath Organization (WHO)

*Correspondence to:
C. Gialdini
cgialdini@crep.org.ar

For peer review only

Abstract

Introduction

Access to comprehensive abortion care could prevent the death of between 13,865 and 38,940 women and the associated morbidity of five million women worldwide. There have been some important improvements in Latin America in terms of laws and policies on abortion. However, the predominant environment is still restricted, and many women, adolescents, and girls still face multiple barriers to exercise their reproductive rights. This research will systematically assess comprehensive abortion policies in five Latin American countries (Argentina, Colombia, Honduras, Mexico, and Uruguay). The aim is to identify barriers, facilitators, and strategies to the implementation of abortion policies, looking at four key dimensions − regulatory framework, abortion policy dynamics, abortion service delivery, and health system and health outcomes indicators − to draw cross-cutting lessons learned to improve current implementation and inform future safe abortion policy development.

Methods and analysis

A mixed-method design will be used in the five countries to address the four dimensions through the Availability, Accessibility, Acceptability, and Quality of Care model. The data collection tools include desk reviews and semi-structured interviews with key actors. Analysis will be performed using thematic analysis and stakeholder analysis. A regional synthesis exercise will be conducted to draw lessons on barriers, facilitators, and the strategies.

Ethics and dissemination

The project has been approved by the World Health Organisation Research Ethics Review Committee (ID: A66023) and by the local research ethics committees. Informed consent will be obtained from participants. Data will be treated with careful attention to protecting privacy and confidentiality. Findings from the study will be disseminated through a multipurpose strategy to target diverse audiences to foster the use of the study findings to inform the public debate agenda and policy implementation at national level. The strategy will include academic, advocacy and policy arenas and actors, including peer-reviewed publication and national and regional dissemination workshops.

Strengths and limitations of this study

- The study encompasses five countries with a variety of legal environments regarding abortion in order to show different contexts while looking at common patterns regarding barriers, facilitators, and strategies.
- From all complex social, legal, cultural, and ethical factors shaping abortion care and policies, this research will focus on four, using the Availability, Accessibility, Acceptability, and Quality of Care framework.
- This study will focus on factors influencing implementation.
- Data will be collected through standardized instruments, which might not reflect the heterogeneity among and within participating countries.

INTRODUCTION

Access to comprehensive evidence-based abortion care could prevent the death of between 13,865 and 38,940 women (1) and the associated morbidity of five million women worldwide. (2) Improving access to quality abortion care is an essential strategy for the provision of universal access to reproductive health and for the achievement of the United Nations Sustainable Development Goals (SDG): good health and well-being and gender inequality (SDGs 3 and 5). Women's access to sexual and reproductive

For peer review only - http://bmjopen.bmj.com/site/about/guidelines.xhtml

rights can also contribute to reproductive empowerment, addressing additional SDGs such as quality education (SDG 4), sanitation and hygiene (SDG 6), decent work and economic growth (SDG 8), reducing inequalities (SDG 10) and fostering peace, justice, and strong institutions (SDG 16). (3)

Since the adoption of the Programme of Action (PoA) of the International Conference on Population and Development (ICPD) 28 years ago, countries have been called upon to strengthen their commitment to women's health by addressing unsafe abortion and supporting a woman's right to decide. (4) Although some improvements in terms of policies on sexual and reproductive health −including law reforms− have been achieved, many women in Latin America face multiple barriers to exercising their reproductive rights. (5)

Obstacles to quality abortion access are a matter of human rights and of public health: in this region, at least 10% of all maternal deaths are a consequence of unsafe abortions and near 760,000 women are hospitalized per year due to complications related to unsafe abortion, which violates their rights to life and health. (6) The obstacles faced by women, adolescents and girls when trying to access quality abortion include the interpretation and the implementation of legal grounds (i.e., legal indications under which is legal to have an abortion), the lack of access to information, stigma, restricted availability of health-care providers and scarcity of facilities that can lawfully provide services, among others. (7) These barriers can disproportionately affect specific groups, such as adolescents, women that are poor, indigenous women, women living in small towns, and other women in vulnerable social conditions.

In this context, reforming abortion laws has proven to be challenging. Most Latin American countries have restrictive abortion laws, allowing legal termination of pregnancy only under certain grounds such as rape, severe fetal impairment or when the woman's life or health is at risk. (8) Abortion is legal without restriction as to reason in the first trimester of pregnancy in Argentina, Cuba, Uruguay, and nine states of Mexico (Mexico City, Oaxaca, Hidalgo, Veracruz, Baja California, Baja California Sur, Sinaola, Guerrero and Colima). Moreover, in February 2022, the Colombian Constitutional Court ruled to decriminalize abortion up to 24 weeks of pregnancy.

For peer review only

Despite a restricted environment, some countries have managed to enhance the availability, accessibility, acceptability and quality of abortion services and care by resorting to alternative strategies. Court judgments and public health guidelines have been used to expand the interpretation of the circumstances under which abortion is allowed. In settings with very restrictive legal environments, like Honduras, guidelines to manage post-abortion care have also been issued. (9)

Taking this regional context into consideration, we will systematically look at comprehensive abortion policies in five Latin American countries −Argentina, Colombia, Honduras, Mexico, and Uruguay− to understand how context, content, actors and processes affect the implementation of comprehensive abortion policies. The aim is to assess abortion regulations[1] and their use in practice, the policy dynamics, the service delivery arrangements, and the abortion-related health indicators and how they are monitored and evaluated, to identify barriers and facilitators to the implementation of comprehensive abortion policy and the strategies used to address them in each country. Finally, we will identify similarities and differences among barriers, facilitators, and strategies of policy implementation across countries, and draw lessons learned from these implementation processes and the strategies to improve access to evidence-based abortion care.

METHODS AND ANALYSIS

Design and framework

In this research study we will look at the context, content, actors, and processes affecting the implementation and monitoring of comprehensive abortion policies and comprises five country-case studies and a regional synthesis of findings. We will use a mixed-method design applying diverse data gathering techniques and analysis strategies to assess four key dimensions of abortion policy[2]:

- The **regulatory framework** (dimension 1)

[1] By Regulations we mean rules or guidelines established and enforced by an authority such as constitutional clauses, laws, decrees, and other sort of administrative regulations

[2] By Policy we mean a system of laws, regulatory measures, courses of action, and funding priorities concerning a given topic promulgated, in this case, abortion.

For peer review only - http://bmjopen.bmj.com/site/about/guidelines.xhtml

- The **policy dynamics** (dimension 2)
- The **service delivery arrangements** (dimension 3)
- The **health system and health outcomes indicators** (dimension 4)

In dimension 1 (regulatory framework) we will assess how and to what extent regulations −both written and in practice− pose as barriers and facilitators to quality abortion access, and the strategies to deal with them. We will address the traits and use of existing abortion related regulations. This dimension encompasses laws, resolutions, decrees, health standards and health guidelines, technical guidelines, and other official documents with or without legal status. First, we will perform a descriptive analysis of the regulatory framework, and then an examination of the practice.

In dimension 2 (policy dynamics) we will assess how aspects such as agenda setting, design and implementation processes, and governance and actors' involvement, pose as barriers and facilitators of abortion policy, and how they are addressed through different strategies. We will assess the agenda setting looking at the political and social conditions that place abortion policy on the public agenda. For design and implementation aspects we will focus on the shaping of content in policy formulation and on the main challenges of implementation. Governance issues include the capacity of health authorities to rule, reinforce and have control of the implementation of abortion policy for the provision and access to comprehensive abortion care. We will address the policy dynamics from the perspective of policy makers, health services managers and women's organizations.

In dimension 3 (service delivery arrangements) we will assess how health services are organised to ensure access to quality abortion care, and how barriers and facilitators are addressed by different strategies. (10-14)

In dimension 4 (health system and health outcomes indicators) we will assess what information on health system and health outcomes indicators related to abortion care are monitored and evaluated. We will address the process of generating, collecting, reporting, monitoring, and evaluating health system and health outcomes indicators, and how this evaluation accounts for policy implementation progress and quality of care oversight.

We will assess these dimensions through availability, accessibility, acceptability, and quality of care as framed in the Availability, Accessibility, Acceptability, and Quality of Care (AAAQ) model. We will use this framework to gather and systematize the data

For peer review only

coming from the two data collection instruments: semi-structured interviews and document review. *Availability* refers to the need of having enough functioning public health and health care facilities, goods, and services. *Accessibility* refers to the need of making all those facilities, goods, and services physically accessible and affordable to everyone within the jurisdiction of the State party without discrimination. *Acceptability* refers to the need of the health system responses to be respectful of the law, medical ethics, and to be culturally appropriate and sensitive to gender and age. *Quality of care* also refers to the need for health goods and services to be scientifically and medically approved and of good quality following WHO standards. (15) Specifically, *quality of abortion care* refers to the fact that the provision of abortion services should be effective, efficient, accessible, acceptable (patient centred), equitable and safe. (1)

As a result of the appraisal of the four dimensions, we will identify the **barriers and facilitators** to availability, accessibility, acceptability, and quality of care regarding abortion related to abortion policy implementation.

Finally, we will synthesize findings from the case studies to draw lessons learned on cross-cutting barriers and facilitators, as well as the similarities and differences on how comprehensive abortion policies are implemented in the participant countries. These lessons learned can be used to inform future decision-making across the region and other sociocultural and geopolitical contexts. (See Fig. 1).

For this study, comprehensive abortion care includes the elements of induced abortion under legal indications as well as all the elements of post abortion care: information, counselling, treatment, contraceptive services, reproductive health services, community, and service providers' partnerships.

Settings

We will conduct case-studies in five selected countries: Argentina, Colombia, Honduras, Mexico, and Uruguay. The selection of settings addresses diversity in abortion regulations as well as diversity in countries' institutional political organization across a continuum of abortion related legal access. We aim to include countries going from an absolute legal restrictive environment with only post-abortion care provision to countries

For peer review only - http://bmjopen.bmj.com/site/about/guidelines.xhtml

with less restrictive legal status of abortion, availability of abortion guidelines, or registration and mifepristone and misoprostol availability, two medicines used in the medical abortion process. The selection also considers common issues on abortion policy implementation and indicators among countries. Country profiles are summarized in Table 1.

Table 1. Country profiles

	Argentina	Colombia	Honduras	Mexico*	Uruguay
**Socio economic profile**	• 42% of the population lives under poverty† • 42, 3 Gini Coefficient‡ •High level of Inflation. The second country with highest (triple digit inflation) •A historically large middle class is shrinking	• 42% of the population lives under poverty† •54, 2 Gini Coefficient‡ •Under a peace agreement process, with still sites with high levels of violence and isolation	• 48% of the population lives under poverty† •48, 2 Gini Coefficient‡ •The size of the middle class was among the smallest in the region (18% compared to an average 41%) •High levels of violence: over 38 homicides per 100,000 inhabitants	• 44% of the population lives under poverty† •45, 4 Gini Coefficient‡ •High levels of social and political violence connected with the illegal drug market	•11% of the population lives under poverty† •40, 3 Gini Coefficient‡ • Its middle class is the largest in the Americas, representing more than 60% of its population‡‡
**Division of the geographical power**	Federalism with shared competences between national and local government	Centralist with local powers	Unitarian	Strong Federalism	Centralist
**Abortion legal model**	Mixed (indications and voluntary interruption of pregnancy) with partial decriminalization	Mixed with total decriminalized up to 24 weeks	Total ban	Mixed at some states <br>Indication model in other states.	Mixed with partial decriminalization
**Main characteristics of abortion regulation**	• The law allows any health professional to provide abortion services • No rape report is required for access legal abortion under the rape indication •There is no abortion committee •Conscientious objection is allowed but with limitations and only at an individual level	• The law allows any health professional to provide abortion services • There is no abortion committee • Ambulatory abortion is accepted • No rape report is required for access legal abortion under the rape indication • Conscientious objection is allowed but with limitations and only at an individual level	----------	• In some states rape report is required • There is no abortion committee • Conscientious objection is allowed but with limitations and only at an individual level at the national level but there is still	• There is an abortion committee •Medical based regulation, with several other requirements • Conscientious objection is allowed in broad terms, both individual and institutional
**Protocol of safe and legal abortion**	Yes National	Yes National	No Just Protocol for Management of Obstetric Complication	Yes Locals with variations	Yes National
**Institutional adjudication**	Congress	Court	----------	Local legislatures	Congress
**Level where care is provided**	First and second level	First and second level	Second level	First and second level	Second level
**Health service arrangements**	Public health sector is the main abortion provider together with the community sector (also called acompañantes or informal health system)	Private clinics (Pro Familia and Orientame) are two of the main abortion providers	Some Public hospital are the ones proving abortion care	Public specialized abortion clinics are the main providers in Mexico City	Public health sector is the main provider, and private clinics are not promoted

* Mexico has different regulation it each state, so the references here are regarding the national level.

For peer review only

† World Bank. Pobreza en América Latina. Datos estadísticos 2022. Available at: https://es.statista.com/temas/8701/pobreza-en-america-latina/#editorsPicks.
‡ World Bank. Desigualdad en la distribución de ingresos basado en el coeficiente Gini en América Latina y el Caribe en 2021, por país. 2021. Available at: https://es.statista.com/estadisticas/1267584/latinoamerica-coeficiente-gini-desigualdad-de-ingresos-por-pais/.
‡‡Word Bank. Uruguay: panorama general. 2023. Available at: https://www.bancomundial.org/es/country/uruguay/overview#:~:text=En%20t%C3%A9rminos%20relativos%2C%20su%20clase,la%20COVID%2D19%20en%202020.

Data collection and tools

We will utilize two data collection methods: desk review (for dimensions 1 and 4) and semi-structured interviews (for dimensions 1, 2, 3, and 4). The data collection process is summarized in Table 2. We will use the quantitative data not only to inform the qualitative process, but to integrate it into the analysis to better elucidate different aspects of the dimensions, thereby allowing for a robust process of triangulation.

Desk review

For dimension 1, we will review the texts of regulations to identify and categorize abortion related regulations, we will structure a data extraction matrix into two domains:
− the abortion regulatory framework, which refers to the specific regulations and practices related to abortion.
− the legal environment, which includes regulations that are not specifically about abortion but have a direct impact on access to abortion health services (e.g., laws regarding patient confidentiality, norms regarding minors' consent, norms on mental or intellectual disabilities, laws related to sexual violence).

For dimension 4, we will conduct a systematic search for country-specific statistics, reports and documents. National official reports and databases containing processes, outputs, or outcome indicators relevant to abortion care at national level will be reviewed and gaps in indicator reporting, monitoring and evaluation will be identified.

We will base the data extraction matrix for dimension 1, on the topics included in the WHO Global Abortion Policies Database (9, 16) and in the report "*Barreras en el acceso a los abortos legales: una mirada a las regulaciones sanitarias que incluyen el uso del misoprostol*". (17) For dimension 4, we will use a data extraction matrix structured with the AAAQ domains to identify and categorize abortion related indicators. From each

For peer review only - http://bmjopen.bmj.com/site/about/guidelines.xhtml

reported indicator, we will collect data on indicator definition, method of measurement, data collection methodology and frequency, date of publication, source of information and indicator value. If data are stratified (e.g., race, gender, age, urban/rural), this information will also be collected.

Semi-structured interviews

We will conduct semi-structured interviews (18) to explore:

- how legal and regulatory instruments are used, how they pose as barriers and facilitators to abortion service provision and access, and how regulations are translated into practice (dimension 1).
- the policy dynamics related to agenda setting, governance issues, financing schemes, and actors' involvement (dimension 2).
- which are the service arrangements adopted according to the different settings, human resources, and the technologies available, and which were the triggers for the model/different models currently used (dimension 3); and
- which approaches each country uses for monitoring abortion-related care and for the oversight of maternal mortality, near miss and quality of care and how monitoring and evaluation of process –and outcomes– indicators related to abortion are operationalized (dimension 4).

An experienced interviewer with social science or public health background with legal or programmatic knowledge will conduct the interviews. The interviewers will obtain informed consent, conduct the interviews in person or through digital technology, and record and transcribe the interviews.

Participants

For the interviews, we will use a purposive sample of twenty key informants for dimensions 1 to 3 and 10 key informants for dimension 4 in each country. We seek to obtain a broad range of perspectives on the subject (19). The selected subjects will be key informants in the abortion policy domain, encompassing different categories: health providers, sexual and reproductive health policy decision makers, researchers, leaders of women's organizations, judicial agents, politicians, opinion leaders, etc.

For peer review only

The sample size is a target overall number, which we will adjust during the data collection process. We consider that twenty key informants for dimension 1 to 3 and ten key informants for dimension 4 in each country is feasible sample size that should allow for variability along the different categories (see Table 1).

The identification of participants is expected to be different depending on how broad or narrow the categories or levels from where they are identified are. The criteria of variability of forums or levels aims at obtaining diversity and having those that yield the most plentiful data in the matter.

We will identify the key informants through a mapping of actors. A first round of mapping will be based on teams' expertise and networks. We expect to easily identify narrow-level participants such as decision makers (who are few in each national abortion domain). For broad categories, such as health providers and activists (who are many and disperse in different settings), we will ultimately define the selection strategy with each country's principal investigator to achieve a convenience sample that accounts for variability. There may be a second round of mapping to select knowledgeable stakeholders identified by the interviewees from the first round.

Table 2. Data collection

	**Dimension 1**	**Dimension 2**	**Dimension 3**	**Dimension 4**	**Total**
**Argentina**	Desk review<br>20 interviews	20 interviews	20 interviews	Desk review<br>10 interviews	2 desk reviews<br>70 interviews
**Colombia**	Desk review<br>20 interviews	20 interviews	20 interviews	Desk review<br>10 interviews	2 desk reviews<br>70 interviews
**Honduras**	Desk review<br>20 interviews	20 interviews	20 interviews	Desk review<br>10 interviews	2 desk reviews<br>70 interviews
**Mexico**	Desk review<br>20 interviews	20 interviews	20 interviews	Desk review<br>10 interviews	2 desk reviews<br>70 interviews
**Uruguay**	Desk review<br>20 interviews	20 interviews	20 interviews	Desk review<br>10 interviews	2 desk reviews<br>70 interviews

For peer review only

**Total (five countries)**	5 desk reviews 100 interviews	100 interviews	100 interviews	5 desk reviews 50 interviews	10 desk reviews 350 interviews

Analysis

Document review data analysis (each country study)

For dimension 1, we will chart and narratively synthesize data from the document review based on the explicit wording and content of the text, following the categories of the WHO Global Abortion Policies Database. (9, 16). This WHO database contains information related to authorization and service-delivery requirements, conscientious objection, penalties, national SRH indicators, and UN Treaty Monitoring Body concluding observations among other documents.

The written words of the documents, however, take their meaning from their purpose and context, and often require interpretation. Therefore, for the interpretation of competing or inconsistent legal primary sources, legal secondary sources will be used. Additionally, we will carry out a preliminary classification as barrier or facilitator for each topic for the purpose to assess them in the interviews.

For dimension 4, we will synthesize data from the document review and further collect indicators for abortion care; we will classify and analyse this data according to the AAAQ domains. The data extraction matrix will also be based on indicators for abortion care as identified as part of the WHO 2022 indicators for abortion, the Danish Institute for Human Rights reports on sexual and reproductive health and rights and the systematic review by Dennis *et.al.* (20-23) We will identify gaps between the extraction matrix and the available indicators.

We will use data from the desk reviews to gain a comprehensive understanding of the subject matter and identify key themes or gaps in knowledge. We will also use country specific statistics and documents to inform the interviews.

Interview data analysis (country studies)

For peer review only

We will use an inductive thematic coding approach with the data from the interviews for each dimension and organize a matrix round central aspects of the corresponding dimension. A content analysis will follow the coding to identify emerged themes and patterns among them. (24) We will identify barriers and facilitators of policy implementation in relation to the AAAQ framework in a data reduction matrix. The first step will be to extract the data from the interviews and place it within the topics of the data extraction matrix. Then, a second reading of the data in the matrix will take place, guided by specific questions for each dimension. Finally, the analysis of barriers, facilitators and strategies will be organized under the AAAQ matrix.

We will conduct a stakeholder analysis to map the actors who have had an interest and have affected or influenced the implementation of abortion policy (dimension 2), to better understand their role in the continuum supporters-blockers, following the questions suggested by Schmeer (25):

- Who are the most important abortion stakeholders (from a power and leadership perspective)?
- What are the different stakeholders' positions on abortion policies?
- What do the different stakeholders see as possible advantages or disadvantages of abortion policies?
- Which stakeholders form alliances? Is there potential for new alliances to form?

<u>Identification and synthesis of barriers, facilitators, and strategies of comprehensive abortion policy implementation (country studies)</u>

We will then apply the findings from dimensions 1 to 4 to a data grid organised with the AAAQ framework to better illustrate the barriers, facilitators, and strategies to deal with them. This aims to group the information from the four dimensions in one single document, which will shed light on strategies used towards comprehensive abortion policy implementation. (Fig.1)

We will analyse this information using substantive themes (or codes or clusters of codes). The rearrangements and recombination may be facilitated by depicting the data graphically or by arranging them in lists, for example.

The collection of quantitative data will provide a quantifiable framework to evaluate barriers and facilitators in abortion care access and quality. We will obtain a holistic view

For peer review only - http://bmjopen.bmj.com/site/about/guidelines.xhtml

by integrating quantitative and qualitative data, addressing both the qualitative depth of policy experiences and the quantitative breadth of their impact on abortion care policy.

Lessons learned from the five country studies (regional synthesis)

At this final stage of the study, we will conduct a regional synthesis exercise to draw lessons on barriers, facilitators and the strategies provided by the five country studies. Once the first version of this synthesis exercise is ready, we will conduct a validation process involving members of all five national teams to reach consensus on the synthesis. For this validation, we will apply an adjusted nominal group technique. (26)

Summary of study process

We expect to start the study in February 2024 and finish it in May 2025. The study process is summarized in Table 3:

Table 3. Study process

	Data collection	Data analysis	Final product
**Argentina**	**D1-D4**<br>2 desk reviews<br>70 interviews	**D1-D4**<br>Barriers and facilitators<br>**Model AAAQ**	**1 country report**
**Colombia**	**D1-D4**<br>2 desk reviews<br>70 interviews	**D1-D4**<br>Barriers and facilitators<br>**Model AAAQ**	**1 country report**
**Honduras**	**D1-D4**<br>2 desk reviews<br>70 interviews	**D1-D4**<br>Barriers and facilitators<br>**Model AAAQ**	**1 country report**
**Mexico**	**D1-D4**<br>2 desk reviews<br>70 interviews	**D1-D4**<br>Barriers and facilitators<br>**Model AAAQ**	**1 country report**
**Uruguay**	**D1-D4**<br>2 desk reviews<br>70 interviews	**D1-D4**<br>Barriers and facilitators<br>**Model AAAQ**	**1 country report**
**Total (five countries)**	**10 desk reviews and 350 interviews**	Barriers and facilitators per dimension in the 5 countries<br>**Model AAAQ**	**5 country reports**
**Regional level**	**	**Synthesis exercise of the 5 country reports**	**1 regional report on lessons learnt**

Positionality statement

For peer review only

We are researchers working in the field of abortion policy implementation, sexual and reproductive rights and evidence-based practices living in Argentina. We will work on making sure that any biases or assumptions we have or we may bring are documented.

<u>Patient and public involvement</u>

None.

ETHICS AND DISSEMINATION

The project has been approved by the World Health Organisation Research Ethics Review Committee (ID: A66023). Moreover, it has been approved by the local Research Ethics Committee in the five participating countries: Centro Rosarino de Estudios Perinatales (Argentina), Profamilia CEIP (Colombia), Universidad Autónoma de Honduras CEIB (Honduras), Instituto Nacional de Salud Pública (Mexico) and Centro Hospitalario Pereira Rossell (Uruguay).

The study will respect the ethical safeguards necessary to obtain informed and voluntary consent and preserve the anonymity of the key informants interviewed. Names of individuals and organizations will not be used in any final or publicly available product resulting from this research. Interviewers will be professionals with proven experience to guarantee ethical principles in conducting the interviews, date and time will be based on the participant's availability.

Data collected in this study will be managed, stored, analysed, and results will be disseminated with careful attention to protecting human subjects' rights to privacy and confidentiality. Specifically, data will be treated in compliance with and approved by the WHO's Research Ethics Review Committee (ERC) and Centro Rosarino de Estudios Perinatales Independent Ethics Committee.

Findings from the study will be disseminated through a multipurpose strategy to target diverse audiences so as to foster the use of the study findings to inform the public debate agenda and policy implementation at national level. The dissemination strategy will include:

-Peer-reviewed publication: Academic article(s) will be submitted to an international journal to disseminate main findings of the comparative analysis of barriers and facilitators of quality abortion policy implementation in the selected countries.

For peer review only - http://bmjopen.bmj.com/site/about/guidelines.xhtml

-National dissemination workshop: this workshop will gather diverse political, academic and policy actors to discuss main findings and discuss barriers and facilitators of quality abortion policy implementation.

-Regional dissemination workshop: this workshop will gather diverse political, academic and policy actors to discuss main findings of the comparative analysis of barriers and facilitators of quality abortion policy implementation in the Latin American region.

Discussion

Latin America countries have several legal and other restrictive conditions regarding abortion. However, some countries of this region also have legal norms that could allow a better access to quality abortion care but are not implemented or are ill-implemented. Interestingly, in the last years, several strategies have been adopted, which have led to improved access to abortion care, such as broad interpretation of existing regulatory frameworks and of policy governance reinforcement, as well as social pressures exerted on decision makers and politicians by women's organizations.

The WHO Global Abortion Policies Database provides a comprehensive compilation of country-specific documents regarding policies and laws. (9, 16) Existing literature has explored abortion policy implementation in various contexts, including a comparative case study that investigated health sector strategies that were useful in expanding or establishing abortion services in six countries around the world. (27) Context and actors involved in abortion reform in some Latin-American countries have also been described, (28, 29) with a recent cross-sectional study characterizing abortion-related complications, management of these complications, and women's experiences with abortion in six countries. (30) A strength of this study is the assessment of different dimensions and actors of abortion policy implementation, which should provide a comprehensive and more precise understanding of the factors that influence the implementation of these types of health policies. Nevertheless, from all complex social, legal, cultural, and ethical factors shaping abortion care and policies, this research will focus on only four to identify relevant barriers and facilitators to abortion policies, using the AAAQ framework.

Another strength of this project is that, as a multi-country study, it encompasses five countries from restrictive to more liberalised environments. This will show different contexts while looking at common patterns regarding barriers, facilitators, and strategies.

For peer review only

This data will be collected through standardized instruments and validated by the teams involved in the project. However, there are challenges in providing a systematic comparison of heterogeneous countries. Moreover, the samples in this study may not reflect the heterogeneity of institutions and the proportionality of the population. To capture this complex scenario in each of the five country-case studies, local interview guides will be adjusted and expanded based on contextual factors.

This study will focus on factors influencing implementation and will not analyse how these factors might correspond to other aspects, such as the evaluation of abortion policies, the estimation of satisfaction with the care provided or the magnitude and severity of abortion-related complications in relation to policy implementation. Future research may consider including these and other aspects.

Finally, principal and country investigators will commit to include junior researchers in the development of the study. By conducting this research project, the coordinating team and the local teams will strengthen their skills, including those of junior researchers, to provide more responsive and sustainable policies that address women's needs and rights.

Contributors

AL, CG, ARM, SR, GC, MR contributed to the conception and design of the study protocol, which was discussed with the rest of the authors. CG, ARM, SR, GC, MR, BC, RGPL and AL agreed on the methodological aspects and participated in developing the protocol, reviewing the draft, and making substantial revisions. CG wrote the original outline. ARM together with CG were responsible of the writing, reviewing, and editing the subsequent versions of the protocol. ARM oversaw developing dimension 1 section. SR was in charge of developing dimension 2. MR was in charge of developing dimension 3. GC and CG were in charge of dimension 4. BC developed and wrote all ethics considerations and prepared the original figures with SR and CG. BC and MVO revised it for intellectual content and provided feedback. CG and ARM led the manuscript writing. CG and ARM, with the help of MVO, coordinated the steps for article development and wrote the draft of the article together. AL, SR, GC, MR and RGPL contributed to manuscript writing and revision. All authors contributed to read and approved the submitted version.

Funding

For peer review only - http://bmjopen.bmj.com/site/about/guidelines.xhtml

This project is funded by the UNDP-UNFPA-UNICEF-WHO-World Bank Special Programme of Research, Development and Research Training in Human Reproduction (HRP), Department of Sexual and Reproductive Health and Research, World Health Organization, 20 Avenue Appia, 1211 Geneva, Switzerland. The content of this article is solely the responsibility of the authors and does not reflect the views of the referred programme.

Competing interests

The authors declare that they have no competing interests.

Acknowledgements

We thank the country principal investigators for their comments to the protocol and contributions regarding country specific information: Edgardo Abalos (Argentina), Rocío Murad (Colombia), Jakeline Álger (Honduras), Karla Berdichevsky (Mexico), Karla Flores (Mexico) y Cecilia Stapff (Uruguay). We also thank Agostina Allori for reviewing dimension 1 and Daniel Giordano for his inputs regarding data management and data security aspects.

Authors' details

1. Centro Rosarino de Estudios Perinatales (CREP), Rosario, Argentina.

2. Centro de Estudios de Estado y Sociedad (CEDES), Buenos Aires, Argentina.

3. Consejo Nacional de Investigaciones Científicas y Tecnológicas (CONICET), Argentina.

4. Centro Latinoamericano de Perinatología/Salud de la Mujer y Reproductiva (CLAP/SMR) of the Pan American Health Organization (PAHO).

5. Department of Reproductive Health and Research, World Health Organization (WHO), Geneva, Switzerland.

References

For peer review only

1. World Health Organization (WHO). Abortion care guideline. 2022. Available at: https://www.who.int/publications/i/item/9789240039483
2. World Health Organization (WHO). WHO Unsafe abortion: global and regional estimates of incidence of unsafe abortion and associated mortality in 2008. 2011. http://apps.who.int/iris/bitstream/handle/10665/44529/9789241501118_eng.pdf;jsessionid=6E4DB04A17643FA2E25D8EC75C51E609?sequence=1
3. Dawson A, Bateson D, Estoesta J, Sullivan E. Towards comprehensive early abortion service delivery in high income countries: insights for improving universal access to abortion in Australia. BMC Health Serv Res. 2016; 16, 612.
4. Abortion Worldwide: 20 Years of Reform. Center for Reproductive Rights. 2014. https://www.reproductiverights.org/sites/crr.civicactions.net/files/documents/20Years_Reform_Report.pdf
5. International Human Rights Law and Abortion in Latin America. Human Rights and Abortion. Human Rights Watch. 2005. https://www.hrw.org/legacy/backgrounder/wrd/wrd0106/wrd0106.pdf
6. Abortion in Latin America and the Caribbean. Fact sheet. Guttmacher Institute. 2018. https://www.guttmacher.org/sites/default/files/factsheet/fb_iaw.pdf
7. Doran F, Nancarrow S. Barriers and facilitators of access to first-trimester abortion services for women in the developed world: a systematic review. Journal of Family Planning and Reproductive Health Care. 2015; 41:170-180.
8. Abortion and Reproductive Rights in Latin America: Implications for democracy. Inter-American Dialogue and the Centre for Reproductive Rights. 2015. https://www.thedialogue.org/wp-content/uploads/2019/07/AbortionandReproductiveRightsinLatinAmerica.pdf
9. Johnson BR Jr, Lavelanet AF, Schlitt S. Global Abortion Policies Database: a new approach to strengthening knowledge on laws, policies,

For peer review only

For peer review only - http://bmjopen.bmj.com/site/about/guidelines.xhtml

and human rights standards. BMC Int Health Hum Rights. 2018;12,18(1),35.

10. Dawson A, Bateson D, Estoesta J, Sullivan E. Towards comprehensive early abortion service delivery in high income countries: insights for improving universal access to abortion in Australia. BMC Health Serv Res. 2016; 16, 612.
11. Rowlands S, Lopez Arregui E. How health services can improve access to abortion. The European Journal of Contraception and Reproductive Health Care. 2016; 21, 1, 1–3
12. Mainey L, O'Mullan C, Reid-Searl K, Taylor A, Baird K. The role of nurses and midwives in the provision of abortion care: A scoping review. Journal of Clinical Nursing. 2020; 29(9-10), 1513–1526.
13. Olavarrieta C, Ganatra B, Sorhaindo A, Karver T, Seuc, A. et al. Nurse versus physician-provision of early medical abortion in Mexico: a randomized controlled non-inferiority trial. Bulletin of the World Health Organization. 2015; 93 (4), 249-258.
14. Ramón Michel A, Romero M, Ramos S, Gonzalez Velez C. Seguimiento de la CIPD en América Latina y el Caribe después de 2014. 2014; DOI 10.13140/RG.2.2.24107.54562.
15. World Health Organization (WHO). Gender, equity and human rights. 2016. https://www.who.int/gender-equity-rights/knowledge/AAAQ.pdf
16. Erdman JN, Johnson BR Jr. Access to knowledge and the Global Abortion Policies Database. Int J Gynaecol Obstet. 2018 ;142(1):120-124.
17. Ramón Michel A. Barreras en el acceso a los abortos legales: una mirada a las regulaciones sanitarias que incluyen el uso del misoprostol. 2013. https://clacaidigital.info/bitstream/handle/123456789/489/Misoprostol.reg.barreras.pdf?sequence=5&isAllowed=y
18. Adams WC. Conducting Semi-Structured Interviews. In: Wholey JS, Harty HP, Newcomer KE, editors. Handbook of Practical Program Evaluation. San Francisco: Jossey - Bass; 2015, p. 492–505.
19. Yin RK. Qualitative research from start to finish. The Guilford Press. 2011.
20. World Health Organization (WHO). Safe Abortion: Technical and Policy Guidance for Health Systems. 2012.

For peer review only

https://www.who.int/reproductivehealth/publications/unsafe_abortion/9789241548434/en
21. World Health Organization (WHO). Sexual health, human rights and the law. 2015. https://apps.who.int/iris/bitstream/handle/10665/175556/9789241564984_eng.pd?sequence=1
22. Kähler L, Villumsen M, Holst Jensen M, Falk Paarup P. AAAQ & Sexual and Reproductive Health and Rights. International Indicators for Availability, Accessibility, Acceptability and Quality. The Danish Institute for Human Rights. 2017. https://www.humanrights.dk/sites/humanrights.dk/files/media/migrated/aaaq-srhr_issue_paper_dihr_2017_english.pdf
23. Dennis A, Blanchard K, Bessenaar T. Identifying indicators for quality abortion care: a systematic literature review. J Fam Plann Reprod Health Care. 2017;43(1):7-15.
24. Vaismoradi, M., Turunen, H. and Bondas, T. Qualitative descriptive study. Nurs Health Sci. 2013, 15: 398-405.
25. Schmeer, K. Policy Toolkit for Strengthening Health Sector Reform. Stakeholder Analysis Guidelines. Chapter 2. Stakeholder analysis at a glance. LAC HSR. 2000.
26. Manera K, Hanson C, Gutman T, Tong A. Consensus Methods: Nominal Group Technique. In: Liamputtong P, editor. Handbook of Research Methods in Health Social Sciences. Singapore: Springer Nature; 2019, 737-750.
27. Chavkin W, Stifani BM, Bridgman-Packer D, Greenberg JM, Favier M. Implementing and expanding safe abortion care: An international comparative case study of six countries. Int J Gynecol Obstet. 2018; 143: 3-11
28. Wood S, Abracinskas L, Correa S, Pecheny M. Reform of abortion law in Uruguay: Context, process and lessons learned. Reprod Health Matters. 2016; 24:102–110
29. Ruibal A. Movement and counter-movement: A history of abortion law reform and the backlash in Colombia 2006–2014. Reprod Health Matters. 2014; 22:42–51.

For peer review only - http://bmjopen.bmj.com/site/about/guidelines.xhtml

30. Romero M, Gomez Ponce de Leon R, Baccaro LF, et al. Abortion related morbidity in six Latin American and Caribbean countries: findings of the WHO/HRP multi-country survey on abortion (MCS-A). BMJ Global Health 2021;6: e005618. doi:10.1136/ bmjgh-2021-005618

FIGURE TITLE

Figure 1. Research framework: dimensions & methods

For peer review only

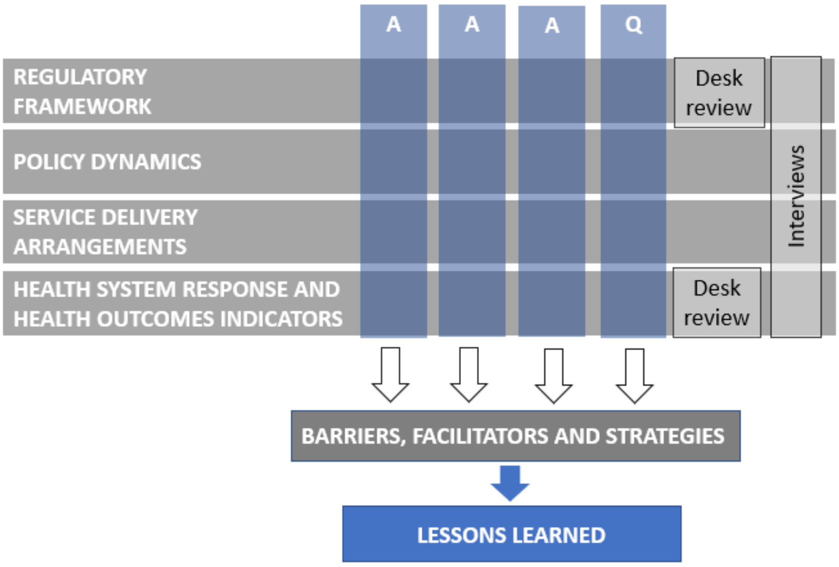

peer review only
